# Rapid differentiation of cystic fibrosis-related bacteria via reagentless atmospheric pressure photoionisation mass spectrometry

**DOI:** 10.1038/s41598-024-66851-y

**Published:** 2024-07-24

**Authors:** Adam Haworth-Duff, Barry L. Smith, Tung-Ting Sham, Cedric Boisdon, Paul Loughnane, Mark Burnley, Daniel B. Hawcutt, Rasmita Raval, Simon Maher

**Affiliations:** 1 Department of Electrical Engineering and Electronics, University of Liverpool, Liverpool, UK; 2 Department of Biochemistry and Systems Biology, University of Liverpool, Liverpool, UK; 3 Department of Women’s and Children’s Health, University of Liverpool, Liverpool, UK; 4 NIHR Alder Hey Clinical Research Facility, Liverpool, UK; 5 Open Innovation Hub for Antimicrobial Surfaces, Department of Chemistry, University of Liverpool, Liverpool, UK

**Keywords:** Analytical chemistry, Bioanalytical chemistry, Mass spectrometry, Medical and clinical diagnostics, Mass spectrometry, Bacteria

## Abstract

Breath analysis is an area of significant interest in medical research as it allows for non-invasive sampling with exceptional potential for disease monitoring and diagnosis. Volatile organic compounds (VOCs) found in breath can offer critical insight into a person’s lifestyle and/or disease/health state. To this end, the development of a rapid, sensitive, cost-effective and potentially portable method for the detection of key compounds in breath would mark a significant advancement. Herein, we have designed, built and tested a novel reagent-less atmospheric pressure photoionisation (APPI) source, coupled with mass spectrometry (MS), utilising a bespoke bias electrode within a custom 3D printed sampling chamber for direct analysis of VOCs. Optimal APPI-MS conditions were identified, including bias voltage, cone voltage and vaporisation temperature. Calibration curves were produced for ethanol, acetone, 2-butanone, ethyl acetate and eucalyptol, yielding R^2^ > 0.99 and limits of detection < 10 pg. As a pre-clinical proof of concept, this method was applied to bacterial headspace samples of *Escherichia coli* (EC), *Pseudomonas aeruginosa* (PSA) and *Staphylococcus aureus* (SA) collected in 1 L Tedlar bags. In particular, PSA and SA are commonly associated with lung infection in cystic fibrosis patients. The headspace samples were classified using principal component analysis with 86.9% of the total variance across the first three components and yielding 100% classification in a blind-sample study. All experiments conducted with the novel APPI arrangement were carried out directly in real-time with low-resolution MS, which opens up exciting possibilities in the future for on-site (e.g., in the clinic) analysis with a portable system.

## Introduction

Cystic fibrosis (CF) is a debilitating genetic condition causing mucus hyper-concentration and decreased mucociliary clearance, leading to chronic lung infections^1^. Early diagnosis and rapid treatment of lung infections in CF patients are critical to decreasing morbidity and extending life expectancy. Diagnosing infections from bacteria such as *Pseudomonas aeruginosa* (PSA) and *Staphylococcus aureus* (SA) can be problematic, particularly in infants, since conventional tests are invasive^2^. For example, induced-sputum testing requires injecting saline solution into the nasal cavity of infants, an unpleasant experience for patients which is frequently mis-administered. Bronchoscopy is highly effective for early diagnosis but requires repeated anaesthesia for CF patients^3^. Less invasive tests, such as lung function measurements, cough swabs, or cough plates, lack sensitivity or reliability. Imaging of lung tissue, including chest X-rays, is effective but relatively expensive and exposes the patient to regular radiation doses^4^. Sputum swabs are a common practice for determining the source of bacterial infections. However, they can often take days to weeks to culture and identify, typically via gram-staining and microscopy.

Rapid identification of bacterial infection is a significant and challenging area of clinical diagnostics and medical research. It might be possible to reduce the unnecessary prescribing of antibiotics if the procedure for the identification of bacteria was quicker and even more helpful if the tests could be carried out non-invasively with no expert training for sample collection. It is, therefore, valuable to develop methods that can discriminate bacteria, such as early-stage infections in CF patients. Ideally, any envisioned non-invasive diagnostic test should have requisite analytical performance whilst also being deployable at the point of care, with minimum risk of harm to patients. Two potentially productive routes to accelerate diagnosis time are readily apparent: direct headspace sampling of bacterial cultures derived from sputum swabs and real-time breath-based assays. Both approaches necessitate online, direct sampling of volatile organic compounds (VOCs) produced by bacteria.

Mass spectrometry (MS) is especially suited to this task, and in recent years, several online or non-invasive MS-based assays have been reported^5^. A wide range of techniques are available for the analysis of VOCs in bacterial headspaces, with methods targeting specific analytes or profiles of VOCs. The gold standard for VOC analysis is widely considered to be gas chromatography-mass spectrometry (GC–MS) for both qualitative and quantitative determination^6,7^. GC–MS has been applied to a wide range of headspace samples^8–10^ and gaseous samples, including breath^8,11^. Chromatographic techniques are powerful but inhibit online/real-time analysis. For instance, GC–MS typically requires extensive sampling stages, such as solid-phase microextraction^11–14^ or thermal desorption^6^. Extensive research efforts have been applied to secondary electrospray ionisation (SESI)^15^, selected ion flow tube (SIFT)^16^ and proton transfer reaction (PTR)^17^ as these can offer on-line analysis of VOCs. SIFT-MS^18^, GC-MS^19,20^, and atmospheric pressure chemical ionisation (APCI)-MS^21^ have all been shown to be capable of determining various bacterial types from direct headspace sampling of cultures with analysis times ranging from 3 to 30 min^18,22,23^. Supplementary Table S1 (supporting information) contains a brief summary of some recent literature regarding bacteria headspace and breath sampling.

As discussed, the fastest conceivable bacterial infection diagnostic assay would be breath-based; this itself is a significant and expanding area of medical research involving non-invasive sampling of continuously available, chemically rich bio-media^24–26^. Breath is primarily composed of O_2_, N_2_, CO_2_, water vapour, volatile organic compounds (VOCs)^6,27^ and non-volatile components^28,29^. Information pertaining to an individual’s health status^30,31^ can be acquired via monitoring disease biomarkers in exhaled breath^25,32^. A wide range of techniques are available for breath analysis, each focusing on distinct components of exhaled breath—certain methods target specific analytes, while others consider patterns of compounds, exhaled breath condensate, and gases, collectively offering complementary insight. By monitoring VOCs in breath, it is possible to augment diagnosis and monitor specific diseases such as diabetes^8,30,33^, asthma^34–36^ and lung disease^37–39^. In-vivo breath analysis as an analytical technique is highly complex, not least due to human physiology and microbiome and difficulties in sampling the end-tidal phase containing low-concentration metabolites of pathological interest.

Electrospray ionisation (ESI) and APCI are amongst the most widely used ionisation techniques in molecular MS. Compared to ESI and APCI, atmospheric pressure photoionisation (APPI) is relatively underutilised. Yet, APPI offers many advantages for VOC analysis. Ionisation of analyte molecules occurs if the ionisation energy (IE) of the analyte molecule is lower than the photon energy emitted from the UV lamp (10.6 eV being the most common). APPI can ionise a broader range of compounds, in terms of molecular polarity, compared to ESI, and is less susceptible to matrix and ion suppression effects than APCI^40,41^. Furthermore, the inability of 10.6 eV lamps to ionise N_2_, O_2_ and CO_2_ directly minimises background interferences, aiding quantification and repeatability. The probability of an ionisation event occurring in APPI is relatively low due to the mismatch in photon flux and the number of analyte molecules present. This is likely further reduced by the simultaneous generation of positive and negative ions that co-exist in the same volume and can lead to some fractional losses due to recombination events.

Photoionisation (PI)-based methods for VOC analysis, including breath, is a growing area of research^42^. Recently, Zhang et al.^43^ achieved good sensitivity detecting SARS-COV-2 infection based on breath VOC profiles, using PI with high-resolution MS (HR-MS) and machine learning (ML). Zhou et al.^44^ modified a commercial APPI source to facilitate breath sampling/analysis^44^; using a high-resolution Q-ToF and collision induced dissociation (CID) they reported the identification of new metabolites in breath. As highlighted by Drabińska et al.^45^, the presence or absence of individual VOCs as disease biomarkers can be misleading and often erroneously assigned. Taking a holistic approach in combination with chemometrics can be advantageous as ion combinations and metabolite fingerprints can be used to readily identify different bacteria. Hundreds of VOCs have been linked to different bacterial strains^18,46,47^, thus, taking a holistic VOC fingerprinting approach is a viable option^22,48,49^.

In this study, we demonstrate the effectiveness of a novel APPI-MS setup. This setup consists of a low-cost, 3D-printed sample delivery system consisting of an APPI lamp, bias electrode, gas delivery ports, and optional liquid dispensing vaporising heater. Traditionally, for APPI analysis, a gaseous dopant molecule (often acetone or toluene) is added to the reagent gas stream to improve sensitivity and enhance detection limits^50^. Dopant ions facilitate charge transfer reactions to ions with greater proton affinity (PA), inducing ionisation in molecules that have higher IE than photons produced by the lamp^51^. Ionisation pathways in APPI broadly follow APCI patterns and have been extensively discussed in many review articles^51–53^. Herein, we demonstrate the applicability of a reagent-less APPI method that is easily accessible for online and direct analysis of VOCs. The method has been developed to enable real-time analysis. Moreover, development has been carried out using a low-resolution mass spectrometer (i.e., with performance metrics akin to a portable system). Following extensive method optimisation and characterisation, bacterial headspace is analysed, specifically SA and PSA cultures demonstrating excellent identification performance (100% classification; blind study of 6 samples), laying the foundation for future clinical investigations concerned with online breath analysis.

## Results and discussion

### APPI-MS interface and novel bias electrode characterisation

**Figure 1:**
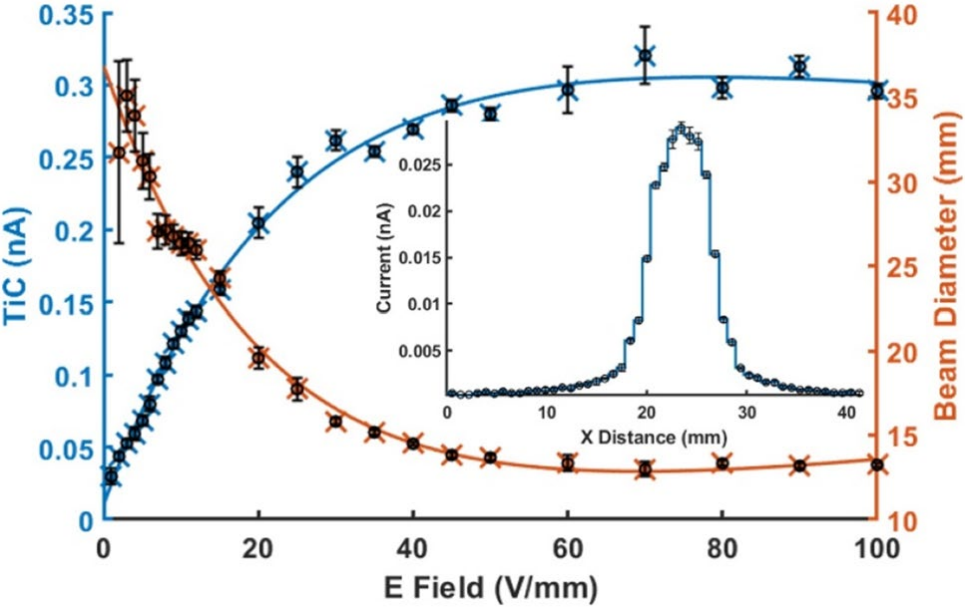
Total ion current (nA) and ion beam diameter (mm) measured by a segmented Faraday detector for an applied electric field between bias electrode and detector from 0 to 100 V/mm. Insert shows ion current hitting individual strips of the segmented ion detector when 50 V/mm field is applied.

An offline characterisation of the ion distribution generated by the UV lamp was performed to aid the design of the APPI-MS interface. Figure 1 depicts the resultant application of a potential bias between the UV lamp and a segmented ion detector. A steady and substantial increase in total measured ion current (cumulative ion current hitting individual detector strips) as electric field intensity increases from 0 V/mm to approximately 50 V/mm is observed, which plateaus for higher field strengths. The increasing force and, therefore, ion velocity due to the increase in applied field yields an order of magnitude increase in ion transmission to the detector. This is likely, at least in part, due to limiting recombination processes between positive and negative ions in the irradiance chamber. Plotting the ion current measured on individual ion detector strips enables visualisation of the ion beam cross-sectional area. Figure 1 insert shows the ion distribution; the ion current is plotted for each strip with the applied voltage between the bias electrode and detector set at 1000 V, corresponding to an electric field intensity of 50 V/mm. The irradiance diameter at maximum ion transmission was approximately 14 mm, decreasing from 35 mm under no potential bias conditions. The diameter of the internal irradiance chamber was, therefore, constructed to be 20 mm to avoid charging the insulating 3D-printed plastic. Some surface charging of the internal wall structure is anticipated, and we expect this will act to further confine the ion cloud diffusion^54^, thereby increasing the quantity of ions sampled by the MS inlet. Furthermore, utilising the smallest irradiance volume possible also reduces neutral analyte diffusion, thereby enhancing ionisation efficiency due to increased analyte concentration.

### Parameter optimisation

To establish the optimal operating conditions, a series of experiments were performed to assess each of the tuneable parameters in the design for a range of compounds related to breath analysis. Ethanol, acetone, ethyl acetate, 2-butanone and eucalyptol were examined. These compounds were selected to encompass a range of VOC parameters: mass range from 47 to 155 u, boiling points from 56 to 176 °C, vapour pressure from 1.9 to 231 mmHg and ionisation energy 9.52–10.48 eV. Additionally, all (except eucalyptol) have previously been reported as significant markers in breath research or disease diagnostics (see supporting information Supplementary Table S2).

### Cone bias voltage

**Figure 2:**
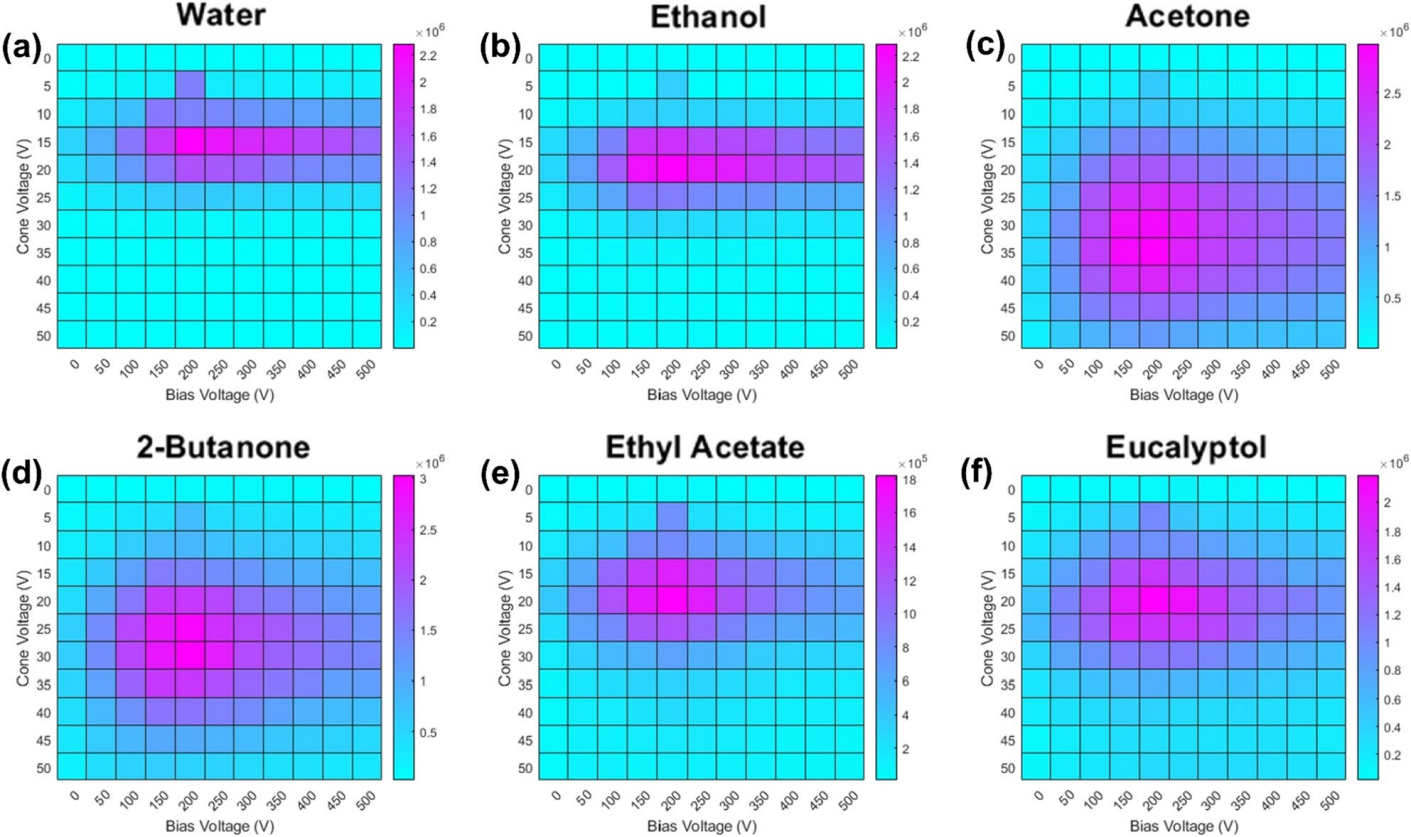
Optimisation heatmaps for (**a**) water, (**b**) ethanol, (**c**) acetone, (**d**) 2-butanone, (**e**) ethyl acetate, and (**f**) eucalyptol, where light blue corresponds to low signal intensity and pink corresponds to high signal intensity, as per the scale bar included in each subfigure. The signal intensity is derived from each analyte’s corresponding protonated molecular ion peak (see supporting information, Fig. S1).

Cone voltage and potential bias electrode are coupled parameters; therefore, they were optimised in tandem. Figure 2 shows the signal intensity heatmaps for each compound examined. Supplementary Figure S1 shows the average mass spectrum from each analyte's maximised bias and cone voltage experiments, with the largest peak in all spectra corresponding to the protonated molecular ion [M + H]^+^. Corresponding tandem MS experiments (Supplementary information Fig. S2) were conducted for the same instrument parameters. Applying potential bias between the lamp and inlet improves the signal intensity by a factor of ~ 10 for all analytes examined. Maximised signal intensity occurs at a bias voltage of 200 V for each compound. It would be of interest to examine higher mass analytes to establish if a broad mass dependency exists, but this is out of scope of this present study. Water (*m*/*z* 37) and ethanol (*m*/*z* 47) both yielded a narrow band of cone voltages that gave relatively high signal intensities from 10 to 20 V and 15 V to 25 V, respectively. Outside of these narrow ranges, the signal intensity dropped significantly. Acetone and 2-butanone also shared a signal intensity response, but instead of bands, in the heat map depiction, they formed concentric circles of increasing signal intensity, peaking at 200 V and 35 V and 200 V and 30 V for bias and cone voltages, respectively. Acetone and 2-butanone showed a higher degree of tolerance towards unoptimised conditions than water or ethanol. Ethyl acetate and eucalyptol exhibited similar concentric circular profiles to acetone and 2-butanone but with a smaller tolerance for unoptimised parameters. Optimum values for bias and cone voltages for ethyl acetate and eucalyptol were both 200 V and 20 V, respectively. Thus, a 200 V bias voltage and 20 V cone voltage were selected for the remainder of the study to give broadly optimal transmission (~ tenfold increase compared to no bias electrode) over the mass range of interest.

### Carrier gas flow rates

The introduction of standards into the APPI chamber is conducted by dosing liquid analytes into an N_2_ carrier gas at precise flow rates using a syringe pump driver (SS Scientific). A 1/16″ stainless steel capillary is concentrically inserted into a ¼″ stainless steel tube and fixed using Swagelok compression fittings. The flow rate of the carrier gas is adjustable via a mechanical variable area flow meter (Brooks Instruments) within the range 0–5 L min^−1^. The liquid solution is dispensed to the end of the capillary, where subsequent nebulisation and transportation to the APPI lamp is facilitated via the carrier gas. A tubular heating element is placed outside of the ¼″ tubing to aid vaporisation of the analyte. No significant carryover is observed when the syringe driver is stopped; after a few seconds, the analyte signal returns to background level.

**Figure 3:**
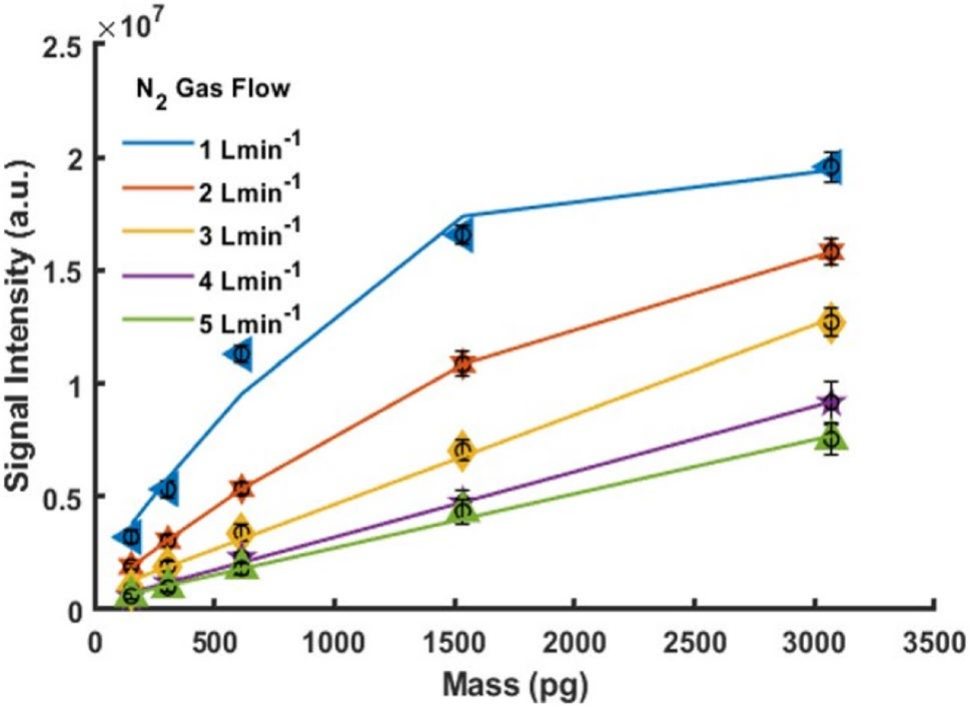
Signal intensity of eucalyptol (*m/z* 155) against analyte amount at a range of nitrogen gas flow rates from 1 to 5 L min^−1^. Data are expressed as mean ± SD (n = 3).

A series of experiments were performed to determine the response of the system to sample and gas flow rate changes for a 20 ppm solution of eucalyptol in water. Figure 3 shows the signal intensity of the [M + H]^+^ protonated molecular ion for eucalyptol for each gas flow rate tested. The amount of eucalyptol introduced into the carrier gas stream was 0.5, 1, 2, 5 and 10 µL min^−1^ corresponding to 15.4, 30.7 61.4 153.5 and 307.0 pg of analyte. The nitrogen gas flow rate was varied between 1 and 5 L min^−1^ in 1 L min^−1^ steps. For carrier gas flow rates above 3 L min^−1^, the signal response was linear across the range investigated (*R*^2^ = 0.996, 0.998 and 0.995 for 3, 4 and 5 L min^−1^, respectively). For 1 and 2 L min^−1^, a reduced upper limit of linearity was observed. The loss in dynamic range is attributed to the higher water vapour concentration in the gas stream. It is well known that increased solvent concentration suppresses analyte signal in APPI due to the absorption of photons by the much higher concentration of solvent^55,56^. The choice of water here was intended to gauge the applicability of the system for future breath analysis, as it contains a relatively high moisture content. Increased signal response for lower carrier gas flow rates is due to a reduced analyte dilution in the carrier gas stream. A carrier gas flow rate of 5 L min^−1^ was used to produce calibration curves, whilst bacteria headspace sampling was carried out using a reduced carrier gas flow rate of 0.2 L min^−1^ to improve sensitivity.

### Vaporisation temperature

**Figure 4:**
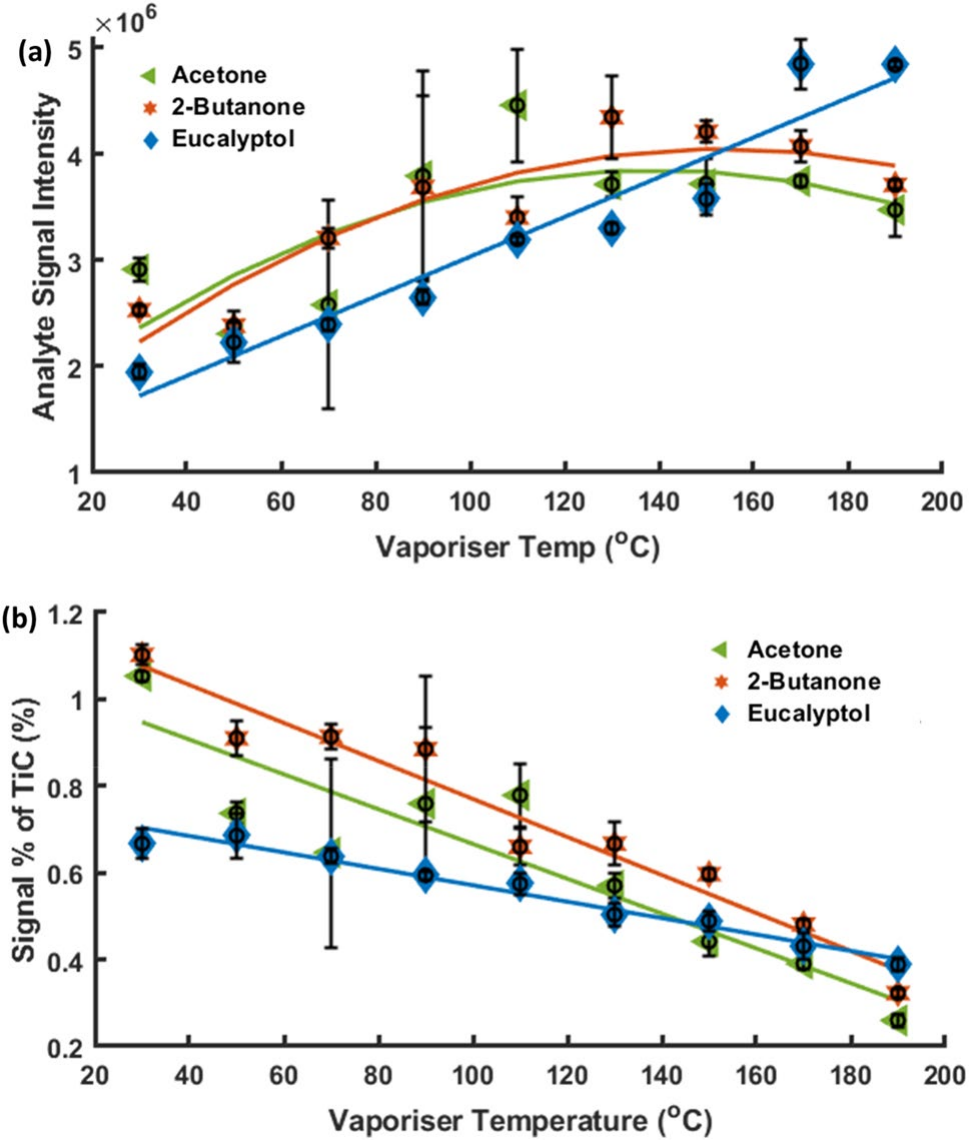
(**a**) Signal intensity of *m/z* 155 (Eucalyptol) against vaporisation temperatures from 30 to 190 °C. (**b**) The peak height for *m/z* 155 is expressed as a percentage of the total ion current (TiC). Data are expressed as mean ± SD (n = 3).

The final element investigated to determine optimal operation was the vaporising heater temperature. A series of experiments were performed by increasing the vaporising heater temperature from 30 to 190 °C in 20 °C steps. 20 ppm solutions of acetone, 2-butanone and eucalyptol in water were individually prepared for optimisation. Each analyte was fed into the capillary, and the temperature was allowed to stabilise before a measurement was initiated. Figure 4a shows the signal intensity of *m*/*z* 59, 73 and 155 peaks relating to acetone, 2-butanone and eucalyptol, respectively, for each temperature set. The intensity values generally increase with increasing temperature for all analytes. Presumably, this is due to more efficient vaporisation of the analyte, which reduces any condensation losses onto the tubing and/or chamber structure. In the case of eucalyptol, this increase continued with increasing temperature, however acetone (b.p. 55.8 °C) and 2-butanone (b.p. 79.5 °C) peaked at ~ 130 °C and ~ 150 °C, respectively, before declining, possibly due to thermal degradation of these analytes. Eucalyptol has the highest boiling point of the three analytes assessed. Whilst increasing the temperature appears advantageous in terms of individual analyte sensitivity, Fig. 4b depicts the relative height of the peaks with respect to the total ion current. It can be observed that increasing the temperature reduces the signal-to-noise ratio of the peaks of interest, possibly due to the other system contaminants being thermally desorbed from the APPI chamber material and gas delivery system. Since the goal of the present study is to determine the metabolite profile of different bacterial samples, the temperature was fixed at 70 °C to avoid the emergence of spurious peaks at the expense of maximising sensitivity.

### Quantification and limits of detection

Calibration curves were produced for each compound diluted in ultra-pure water. Individually, each analyte (ethanol, acetone, 2-butanone, ethyl acetate and eucalyptol) produced a highly linear calibration curve with a coefficient of determination ≥ 0.99 (supplementary information, Table S2). The calibration curves can be found in supplementary information Fig. S3; ethanol produced a linear response between 31 and 315 ppbv, acetone and 2-butanone gave linear responses between 2 and 25 ppbv, ethyl acetate and eucalyptol also gave linear responses between 3 and 36 ppbv.

Ethanol can be found in breath due to bacterial activity in the gut^57,58^, with expected concentrations between 10 and 1000 ppb in healthy breath. Ethanol’s presence can also give information about lifestyle, such as recent consumption of alcohol. Acetone is another VOC naturally found in breath at approximately 1–1000 ppbv^58^ with elevated concentrations greater than 1800 ppbv corresponding to patients with diabetes mellitus^8,33^. In isolation, acetone detection is permissible with a limit of detection (LOD) of 6.8 ppbv and a limit of quantification (LOQ) of 27.8 ppbv. The APPI-MS method is also suitable for the detection of 2-butanone with a LOD of 1.6 ppbv and LOQ of 6.5 ppbv, which is normally present in the breath of healthy people at approximately 20 ppbv^27,59^ and is found in the headspace of bacteria samples of *Pseudomonas aeruginosa*^46^. Some have suggested ethyl acetate is a potential marker related to lung disease^60^. When analysing the breath of these patients, ethyl acetate may be present in concentrations up to 120 ppbv^27^, which is undetectable in the breath of a healthy person. Our method is suitable for ethyl acetate analysis with a LOD of 0.7 ppbv and an LOQ of 5.0 ppbv. Finally, eucalyptol was detectable at 0.9 ppbv and quantifiable at 4.8 ppbv. Eucalyptol was included for future reference, as it is not expected to be found in breath naturally but can be found if an individual has recently consumed mint.

### Bacterial culture classification

**Figure 5:**
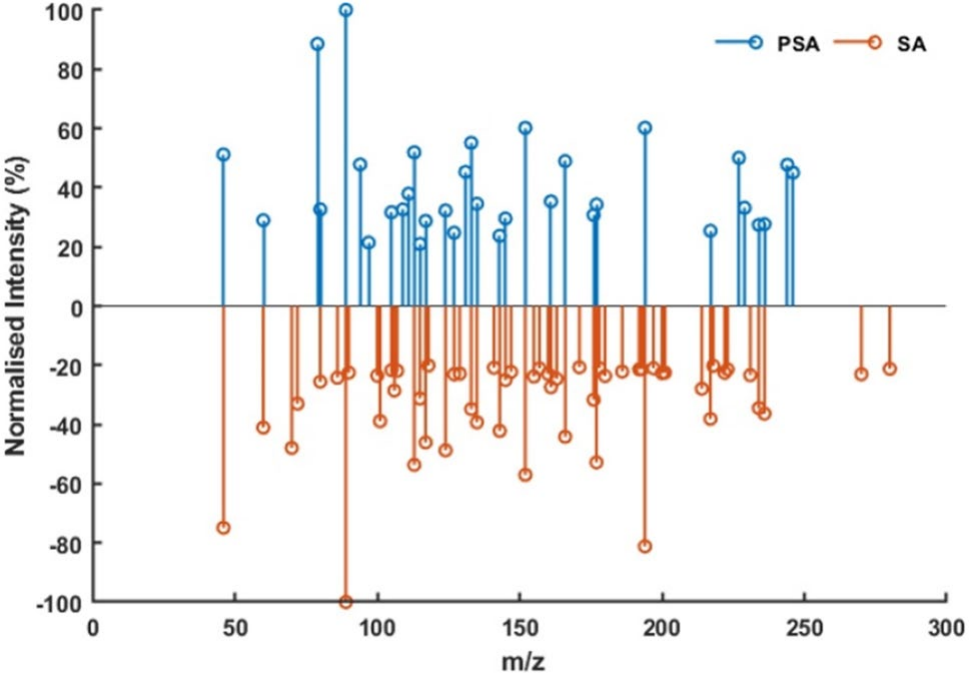
Centroided mass spectra showing all peaks with relative intensities > 20% for PSA and SA bacterial headspace samples.

To assess the suitability of the apparatus for potentially determining bacterial infections in CF patients, PSA and SA cultures were prepared and sampled using 1 L Tedlar bags as described in the methods section. A further cautionary note on Tedlar bag suitability for direct analysis, such as ambient ionisation techniques, can be found in the supplementary information. The resultant collected headspace was evacuated from the bag and passed through the APPI chamber by pumping via a small diaphragm pump. Samples were collected and analysed in batches of four over a three-week period. A new culture was initiated each Monday, and samples were collected on the subsequent Friday; in total, 12 of each type of bacterial headspace were sampled. *Escherichia coli* (EC), unrelated to CF, was included as a control and a means to improve the robustness of the classification. Figure 5 shows centroided spectra for one PSA sample and one SA sample; only peaks with relative intensities above 20% were retained for display purposes. Immediately obvious are a number of distinct visual differences between samples, with a significant number of peaks appearing in only one of the samples. This gives confidence that a classification model can successfully be applied to the dataset. The total ion chromatograms and time indexes of extracted spectra for all samples are shown in Supplementary Figs. S4–6.

**Figure 6:**
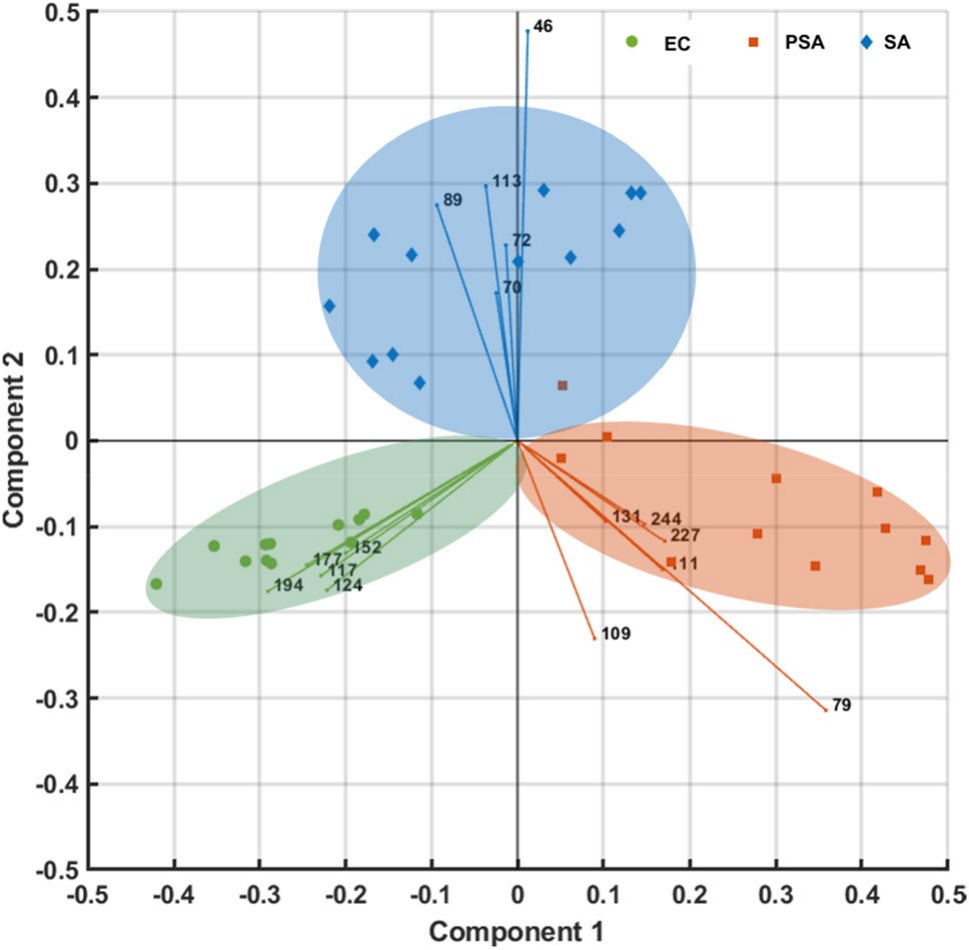
PCA biplot for 12 replicates of 3 types of bacterial culture samples (the numbers are the *m/z* ion peaks that contributed the most in each class for the classification model).

After pre-processing spectral data as outlined in the methods section, 247 peaks were found across all samples and included in a principal component analysis (PCA) model. A data table containing 247 dependent variables and 36 observations was collated. PCA was compiled to visualise spectral differences and dimensionally reduce the dataset. The first three principal components accounted for 86.9% of the total variance, which is an excellent result. A PCA biplot can be seen in Fig. 6, displaying excellent separation and grouping of sample classes with clear class boundaries evident for all 3 groups. Principal component (PC)1 is the discriminating component for EC and PSA, whilst PC2 was responsible for the separation of SA. The remaining PCs (not shown) were not found to differentiate between the sample classes.

Following PCA, a linear discriminant classification model was built using the 247 features. 10 times cross-validation was used to avoid overfitting and improve robustness. 100% of samples were correctly classified by the model. This was a very pleasing and significant result since the samples included multiple cultures, with sample collection compiled and analysed over several weeks. A further set of three SA and three PSA headspace samples was acquired from a fresh batch of cultures a week later. Class predictions were made using the model generated from the training data set in a blind study. 100% of the blind study samples were correctly classified. The results are comparable to that of current SESI-MS and SIFT-MS methods^18,48^. The confusion matrix showing classification results is shown in Supplementary Fig. S7. Headspace samples were also collected for un-inoculated broth samples, in which 200 mL of broth was sampled using the same process as the one described for the bacteria. The results were then plotted on a PCA scatter plot, which can be seen in Supplementary Fig. S8 showing clear separation. Since our laboratory is not designated to handle Class 2 bacterial cultures, it was not possible to conduct online sampling/analysis. Therefore, non-ideal Tedlar bag sampling was used to collect the headspace (Supplementary Figs. S9 and S10). Presumably, even better results could have been achieved with direct headspace sampling.

### Direct (and indirect) breath sampling

**Figure 7:**
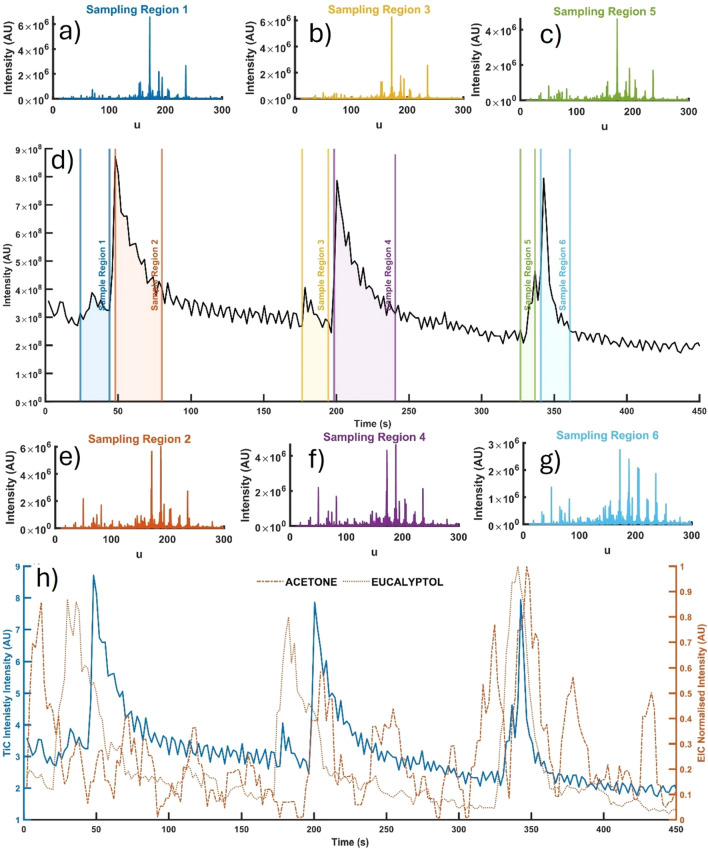
Direct breath analysis. Mean mass spectra are shown in (**a**–**c**,**e**–**g**) which correspond to sampling regions 1 to 6 from the total ion chromatogram (TIC) depicted in sub-figure (**d**). The TIC in (**d**) covers a timeframe of 450 s with 3 breath sampling windows (regions 1, 3 and 5). (**h**) Extracted ion chromatograms (i.e., selected ion monitoring) of *m*/*z* 59 and *m*/*z* 155 corresponding to suspected analytes acetone and eucalyptol, respectively.

The ultimate goal of this work, beyond this present study, is to develop a portable method capable of determining bacterial infection type directly from patient breath. As such, a preliminary study was performed to demonstrate the applicability of the sampling interface for direct (and indirect) breath sampling. Modifications to the sampling apparatus to accommodate Tedlar bag sampling and direct breath injection are shown and outlined in Supplementary Fig. S11. In the first instance, 1 L Tedlar bags were used to collect a single breath from healthy volunteer breath samples before and after consumption of mint-flavoured chewing gum. Supplementary Figure S12 shows the corresponding mass spectra for breath pre- and post-consumption of mint-flavoured chewing gum. Immediately evident is the total increase in the number of relatively intense peaks, including the appearance of *m*/*z* 155 (eucalyptol) as the dominant spectral peak in the post-mint consumption breath. A subsequent experiment was then performed directly analysing breath after consuming mint-flavoured chewing gum. The interface was modified by removing the diaphragm pump used to evacuate the Tedlar bags with a replacement mouthpiece. In the case of direct breath analysis several interesting aspects are evident (Fig. 7). Similar to indirect analysis, the abundance of high-intensity peaks within the spectra increases along with distinct changes in the total ion chromatogram (TIC) corresponding to the breath injection time. 3 breaths were recorded in a single data file; this can be seen in Fig. 7, in which the start of each breath corresponds with the start of sampling regions 1, 3 and 5. Sampling region 1 marks the beginning of the breath sample, in which a single breath was expired into the system; a small but noticeable change to the TIC plot is recorded. However, compared to sampling region 2, the spike in the TIC plot is relatively small, likely due to the high moisture content in breath, which absorbs UV energy and subsequently reduces the ionisation efficiency. The breath signal persists for some 15–20 s after exhalation (regions 2, 4 and 6) since the direct breath sampling (Supplementary Fig. S11c) does not include an active flow through the APPI interface. Evident on the mean spectra from sampling region 2 is a small increase in the number of high abundance peaks and a significant increase in the low abundance peaks throughout the mass range of 10–300 u. By extracting the ion chromatograms for *m*/*z* 155 (eucalyptol) and *m*/*z* 59 (acetone), we can see the eucalyptol peak corresponds with the peak of sampling region one whilst *m*/*z* 59 largely correlates with the second region TIC peak. This would largely be expected since the eucalyptol flavour resides in the mouth, whilst acetone is endogenous and would appear later in the breath cycle. This pattern was evident and broadly repeatable for 3 breath cycles.

Whilst the preliminary breath data shows promise, several challenges must be overcome if this work is to be translated for clinical breath analysis, including developing a standardised breath sampling methodology. Future work will further develop the breath sampling method, investigating the addition of flow controllers and gas sensors to help control and monitor the phases of breath sampled. Nevertheless, with a low-resolution mass spectrometer and without requiring tandem mass analysis, our method correctly identifies SA and PSA in real-time and directly from the headspace of bacteria samples without requiring any reagents. This shows the suitability of our approach for in-vitro bacteria culture analysis and its potential to be extended for online breath monitoring, whereby a holistic analytical strategy offers an attractive prospect for detecting and identifying bacteria.

## Conclusions

In this study, we have designed a novel APPI-MS approach to facilitate non-invasive, real-time, and direct headspace sampling to accurately detect and identify bacterial cultures relevant to cystic fibrosis (CF) infection. Preliminary breath measurements were conducted utilising mint-infused healthy volunteer breath to ascertain the suitability of the APPI-MS interface to sample breath directly. A thorough investigation and characterisation of the new sampling apparatus and methodology have been carried out. We conducted extensive optimisations using a range of compounds that are relevant to breath analysis, establishing limits of detection and quantification in concentration ranges that are of medical interest. Testing was performed directly on the headspace in real time using reagent-free APPI. A classification study was conducted using bacterial headspace from *Pseudomonas aeruginosa* and *Staphylococcus aureus* cultures (including *Escherichia coli*), two prominent sources of bacterial infection in CF patients. Excellent separation and grouping were achieved in PCA space, with 100% classification accuracy for a small blind study (of 6 samples), using a low-resolution mass spectrometer in full MS mode (i.e., without requiring tandem capability) - demonstrating the possibility of carrying out breath analysis in-clinic with a portable (low-resolution) mass spectrometer, which is the subject of future work.

## Methods

### MS settings

All experiments were performed on a Waters Xevo triple quadrupole mass spectrometer (TQ-MS); a low-resolution mass spectrometer (mass resolution ~ 0.4 u, FWHM) released in 2007. As our long-term goal is to develop a method suitable for real-time in-clinic breath analysis, it is necessary to develop our approach on a mass spectrometer with similar performance to a portable mass spectrometer.

Full scan mode was used with a mass acceptance window of 20–300 u, and the scan acquisition time was set to 2 s per scan unless otherwise stated. APPI is an ambient ionisation method^61–66^. Modifications were made to the instrument front-end to make efficient use of the available hardware and to minimise the peripheral equipment needed to operate the bespoke APPI chamber. Tapped threads were drilled into the gas entry ports, which usually supply N_2_ gas to the commercial ESI source. Push-fit pneumatic connectors were inserted into the threaded taps to connect a variable area flow meter whereby the user can manually set a carrier gas flow rate. N_2_ was supplied by a nitrogen generator (Peak Scientific, Glasgow, United Kingdom) or from a gas cylinder. A custom cable (LEMO, Écublens, Switzerland) was constructed to connect the bias electrode to the HV power supply available on the front of the instrument. This enables software selection of the bias potential via the MS tune page in MassLynx (Waters, Wilmslow, UK). Finally, the interlock to prevent operation without the ESI front end in place was overcome by fixing a small metal pin to depress the microswitch. Apart from the stated modifications and unless otherwise stated, the instrument was operated as per the manufacturer’s recommendations.

Tandem-MS analysis was conducted using collision-induced dissociation for certain chemical standards (Supplementary Fig. S2). The collision gas flow and collision energy for the 6 analytes were as follows: 012 ml/min and 7 eV (water), 0.1 ml/min and 10 eV (ethanol), 0.2 ml/min and 23 eV (acetone), 0.2 ml/min and 18 eV (2-butanone), 0.2 ml/min and 10 eV (ethyl acetate), 0.2 ml/min and 13 eV (eucalyptol).

### Chemicals and reagents

A range of chemicals were purchased for analysis, including ethanol (GC standard grade), acetone (> 99.8% HPLC grade), ethyl acetate (99.7% HPLC grade) and eucalyptol (99%) purchased from Sigma Aldrich, and 2-butanone (≥ 99%) from VWR. The VOC standards were diluted in ultra-pure water (v/v) from a Direct-Q 3UV ultra-pure (type 1) water system (18.2 MΩ).

### APPI interface

**Figure 8:**
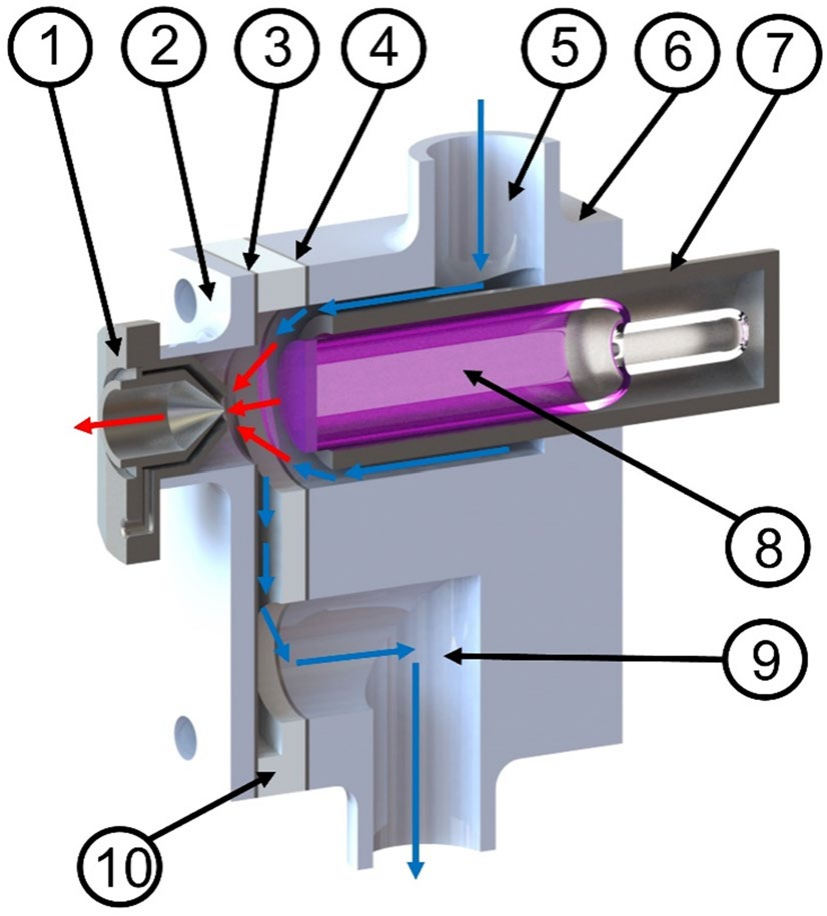
APPI sampling chamber; (1) MS inlet, (2) 3D printed end plate, (3) 2nd electrode, (4) 1st electrode, (5) sample entry, (6) 3D-printed body, (7) grounded metal collar for lamp, (8) UV lamp at 10.6 eV, (9) exhaust, (10) PTFE electrode spacer. Blue arrows indicate the direction of sample gas flow. Red arrows indicate the direction of travel of photo generated ions.

Figure 8 shows a cross-section of the inner structure of the APPI interface. The UV lamp (8) and driver electronics were harnessed from a commercial APPI source (ThermoFisher). The grounded metal collar (7) surrounding the lamp (8) forms a gas tight seal with a bespoke 3D printed enclosure (6). A photopolymer resin material was used rather than the more common extruded (fused deposition) plastic type due to better outgassing characteristics of UV-cured resin when under UV irradiation (from the APPI lamp). The lamp is inserted so that the front face of the lamp aligns with the back edge of the 3D printed chamber. Here, a thin (0.4 mm) metal electrode (4) is located to apply a positive potential bias, with respect to the inlet (1), to confer positive ions created in the ionisation chamber a drift velocity in the direction of the MS inlet. PTFE spacers (10) with holes drilled to enable gas entry and exit provide an ionisation volume directly in front of the MS inlet. A second thin electrode (3) provides the reference potential for the bias voltage and is electrically connected to the sampling cone of the mass spectrometer. Finally, a 3D printed piece (2) push-fits over the sampling cone, and threaded rods/nuts compress each element into a gas tight sampling chamber. It should be noted that the final 3D printed piece can be readily designed to fit onto any mass spectrometer atmospheric pressure interface (API) by changing the diameter of the exit orifice to suit. Rubber bungs with stainless steel and PTFE tubing provide gas entry (5) and exit (9) from the source via either pressurisation from the inlet region or evacuation by a small diaphragm pump at the exit. A heater unit also resides in line with the gas entry (5), which is depicted in supporting Fig. S11a.

### Source characterisation

An offline measurement of the ion distribution generated from the UV lamp was performed. A stainless-steel electrode was fixed 20 mm from a segmented Faraday plate detector (designed and built in-house)^67^. The sensor contains fifty, 0.85 mm wide × 15 mm long, sensing elements capable of simultaneously integrating the ion current striking each strip of the detector. A 12 mm diameter opening was cut into the bias electrode to enable the UV lamp insertion. A voltage applied to the biasing electrode was scanned between 0 and 2000 V in 100 V steps. After each new applied voltage, the ion current hitting each of the 50 strips on the detector was measured and recorded.

### Bacteria sampling

The bacteria were initially grown on agar gel, in which 1 mL of the culture was transferred to 200 mL of LB broth (MILLER) purchased from Merck, in a 1 L conical flask with a gauze bung and left on a shaker bed at 220 rpm in a temperature controlled dark room (37 °C). The bacteria were incubated overnight. After incubation, bacterial headspaces were actively sampled by placing stainless steel tubing into the conical flask and removing gas phase constituents via a peristaltic pump with verderprene tubing for 2 min at a rate of approximately 400 mL/min. 1 L Tedlar bags were used for collection and transferred to the mass spectrometry laboratory for analysis within 1 h after sampling. All bacteria analyses were conducted over a 3-week period in total.

After collection, each sample was evacuated with the APPI-MS interface using a pump set at 400 mL/min over a time period of 5-min (Supplementary Fig. S11b). At the same time, corresponding data files were generated on the mass spectrometer and processed accordingly to yield average spectra for each sample.

### Breath analysis

1 L Tedlar bags were used to collect breath samples for indirect analysis. Similar to sampling the bacterial headspace samples, a pump was used to evacuate the collected sample through the interface set at 400 mL/min (Supplementary Fig. S11b). A data file was started prior to the sample's evacuation and ran until the bag was empty and the TIC had returned to the baseline.

In addition to the collected Tedlar bag samples, direct breath analysis was performed in a rudimentary way (Supplementary Fig. S11c). For direct breath analysis, the participant performed an elongated exhalation over 20–25 s. The tubing used to introduce the aerosolised standards was replaced with a 30 cm length of sterile Tygon tubing. The participant breathed directly through the tubing into the interface, without any active flow assistance. A data file was started prior to the first breath, with multiple breaths recorded.

### Data processing

Raw data collected from each sample were stored in separate MassLynx files, which were named based on method parameters, sample, and repeat information. Conversion from MassLynx.RAW to .mzXML was completed prior to data processing and analysis in MATLAB R2023a (Mathworks). A series of pre-processing steps were performed on the dataset prior to use in classification models^68,69^. Briefly, after mzXML files are extracted into a MATLAB data table, the retention times and total ion currents (TIC) are extracted and searched for abrupt changes in TIC current, indicating the beginning and end of sampling the contents of a Tedlar bag. 5 scans were removed at the beginning and end for every sample to discount any variation during the transient periods. The scans between the two time indexes generated by the MATLAB function findchangepoints() were averaged into a single scan. The intensity information is smoothed and resampled over a uniform 0.1 u grid and normalised. MATLAB function findpeaks() is used to extract the peak height and location information. To avoid multiple peaks sampled with small *m*/*z* shifts affecting classification, the location of each peak was rounded to the nearest integer. The peak information was located in a single table, and any missing peaks not generated by the find peaks algorithm across samples were assigned the value 0. Furthermore, a threshold of 0.5% was applied to the data, and any peaks below the threshold were assigned a 0 value.

### Ethics declarations

This study was approved by the Central University Research Ethics Committee at the University of Liverpool for “A Real-Time Method for Breath Analysis of Healthy Volunteers Using Ambient Pressure Photoionisation Mass Spectrometry (APPI-MS)”, Ref No. 12535.

### Supplementary Information

Supplementary Information.

## Data Availability

All data generated or analysed during this study are included in this published article and its supplementary information files.
