# Supplementary material for: Rapid differentiation of cystic fibrosis-related bacteria via reagentless atmospheric pressure photoionisation mass spectrometry
Source: Sci Rep. 2024 Jul 24;14:17067. doi: 10.1038/s41598-024-66851-y (PMC11269582; doi:10.1038/s41598-024-66851-y)
Supplement: Supplementary file 1 — Supplementary Information. [file 41598_2024_66851_MOESM1_ESM.pdf]

Supplementary Information

Rapid Differentiation of Cystic Fibrosis-Related Bacteria via Reagentless Atmospheric Pressure Photoionisation Mass Spectrometry

Adam Haworth-Duff[1], Barry L. Smith[1], Tung-Ting Sham[1], Cedric Boisdon[1], Paul Loughnane[2], Mark Burnley[1], Daniel B. Hawcutt[3,4], Rasmita Raval[5] & Simon Maher[1]*

[1]Department of Electrical Engineering and Electronics, University of Liverpool, UK,

[2]Department of Biochemistry & Systems Biology, University of Liverpool, UK,

[3]Department of Women's and Children's Health, University of Liverpool, UK,

[4]NIHR Alder Hey Clinical Research Facility, Liverpool, UK,

[5]Open Innovation Hub for Antimicrobial Surfaces, Department of Chemistry, University of Liverpool, UK.

*email: s.maher@liverpool.ac.uk

Contents

Figure S1 .... S3
Figure S2 .... S5
Figure S3 .... S6
Figure S4 .... S6
Figure S5 .... S7
Figure S6 .... S8
Figure S7 .... S9
Figure S8 .... S10
Tedlar Bags: A Cautionary Note for Direct Analysis S11
Figure S9 .... S12
Figure S10 .... S13
Figure S11 .... S14
Figure S12 .... S14
Table S1 .... S15
Table S2 .... S17
Supplementary References S18

Figure S1

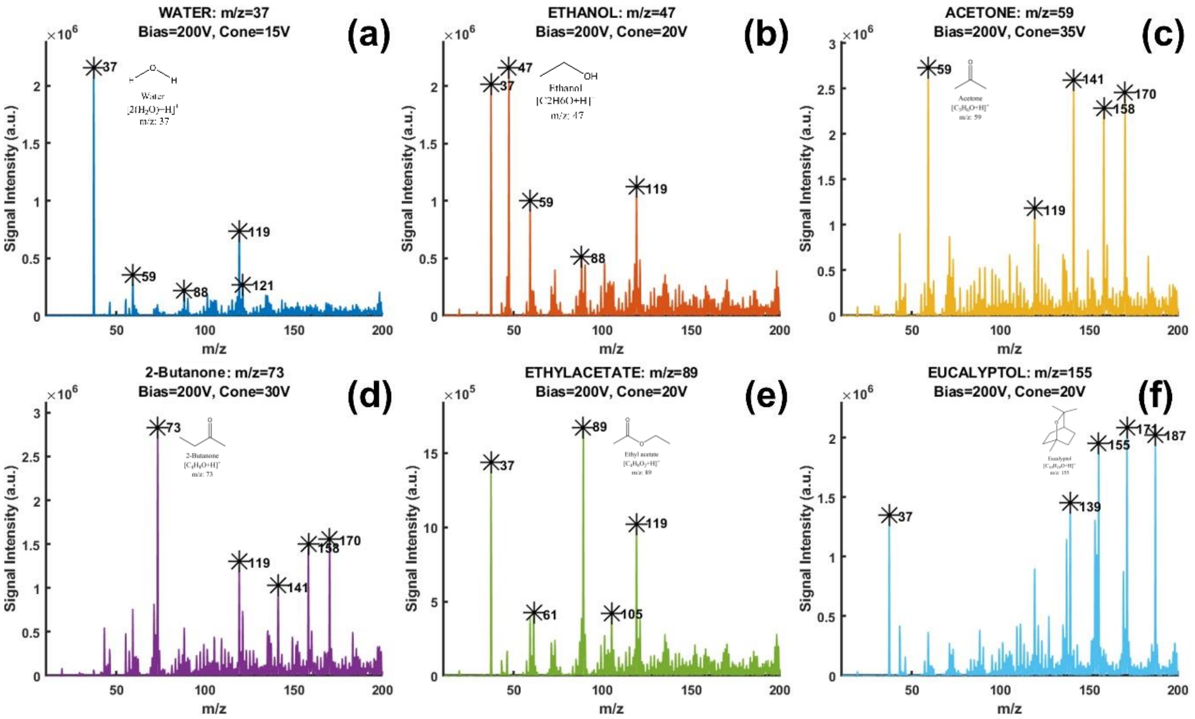

Figure S1: Average mass spectra for (a) water only, (b) ethanol, (c) acetone, (d) butanone, (e) ethyl acetate and (f) eucalyptol. Asterisk (*) peak notation relates to the 5 highest peak intensities.

The top five intense peaks in each averaged spectra have been labelled with an asterisk (*). Protonated ions of each analyte gave the largest peak intensity. Figure S1 (a) shows the average mass spectra for water in which the $[H_3O]^+\cdot H_2O$ peak can be seen at *m/z* 37. Four other peaks above the baseline intensity were also identified at *m/z* 59, 88, 119 and 121 and were noted as background contaminants, probably due to the polymer resin used for 3-D printing structural elements of the interface. Draft photopolymer resin was selected due to its availability, suitability for prototyping and stability once cured; however, as a plastic element, its outgassing characteristics cannot be guaranteed. For the purpose of a pre-clinical prototype, the relatively low intensity of these peaks indicates that these extraneous contaminants can be safely ignored. Figure S1 (b) shows the effect of ethanol addition into the system; the largest peak at *m/z* 47 is expected to be a protonated molecular cation, [M+H]+, from ethanol. Figure S1 (c) shows the average mass spectra for acetone, in which the protonated molecular cation, $[M+H]^+$, can be seen at *m/z* 59. The well-known dopant effect due to acetone presence is readily observed by the occurrence of additional high intensity peaks at *m/z* 141, 158 and 170 and a corresponding decrease in the water peak intensity caused by proton transfer reactions facilitated by acetone. Figure S1 (d) shows the 2-butanone result and the protonated molecular cation at *m/z* 73 as the dominant peak in the spectrum. Interestingly 2-butanone (Proton affinity (PA): 827.3kJ/mol) shows a similar dopant trait as acetone (PA: 812.0kJ/mol) but with reduced efficiency since the contaminant peaks are approximately 40% lower intensity. Figure S1 (e) shows the ethyl acetate (MW 88.11) mass spectral result, with *m/z* 89 as the most intense peak. The ionisation efficiency for ethyl acetate (IE: 10.01eV) is reduced; ~1.6e^5 signal intensity compared to > 2e^6 for other analytes studied. The acetone-doped contaminant peaks are also eliminated from the spectrum, and only *m/z* 37 (water) and *m/z* 119 are present as intense peaks. Figure S1 (f) is for eucalyptol, which responds differently to ionisation by APPI than would be considered normal. A protonated molecular ion peak (*m/z* 155) is present in high abundance >2e^6 but is accompanied by other ion

peaks at similar intensity (m/z 171 and 187). Tentative assignments of these additional peaks in the eucalyptol analysis are suspected as: m/z 187 corresponding to dihydroxy-1,8-cineole (MW 186.25), m/z 171 is likely to be the metabolite hydroxy-1,8-cineole (MW: 170.25) and m/z 153 most likely corresponds to that of 2,3-dehydro-1,8-cineole (MW: 152.23).[1,2]

Figure S2

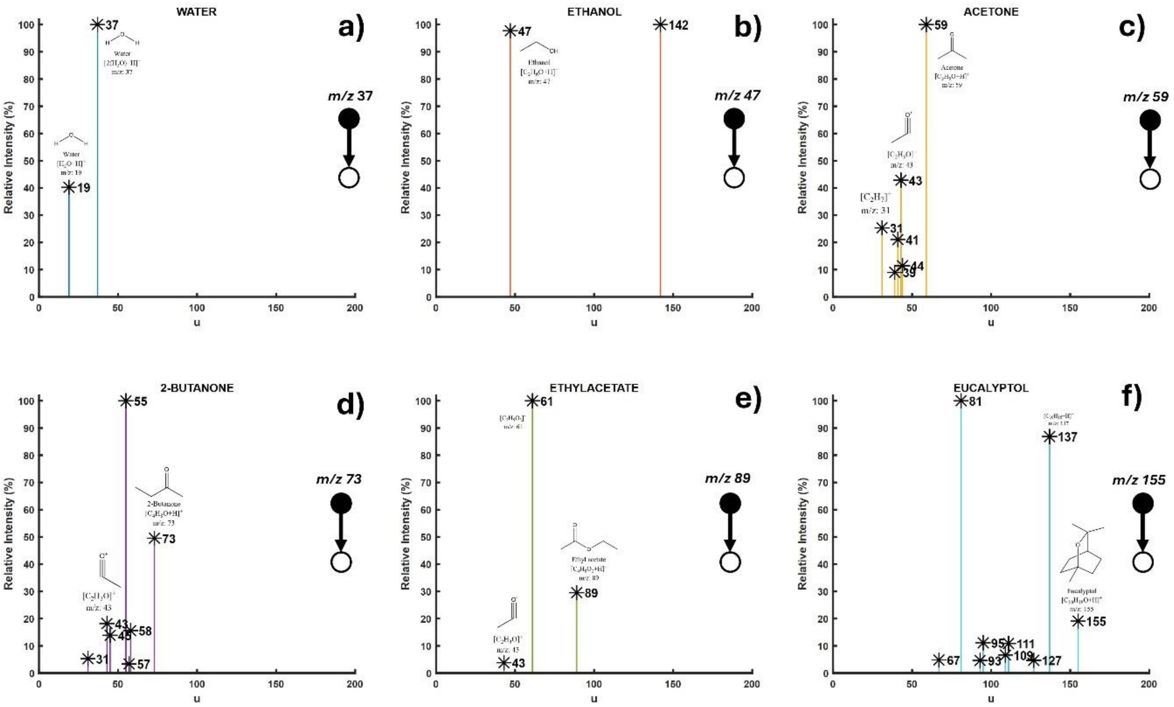

Figure S2: Average tandem-MS mass spectra for (a) water only, (b) ethanol, (c) acetone, (d) butanone, (e) ethyl acetate and (f) eucalyptol. Asterisk (*) peak notation relates to the highest peak intensities.

Figure S3

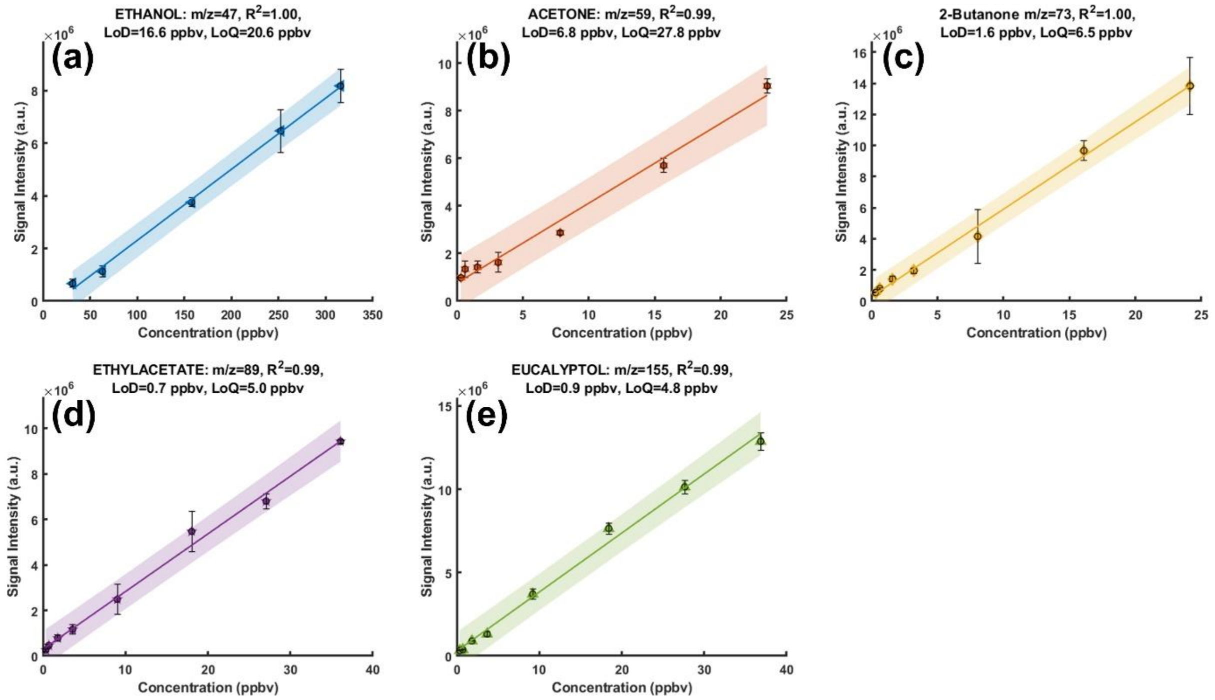

Figure S3: Calibration curves for (a) ethanol, (b) acetone, (c) 2-butanone, (d) ethyl acetate and (e) eucalyptol. Each point on the calibration curves was taken in triplicate with error bars and 95% confidence intervals shown.

Figure S4

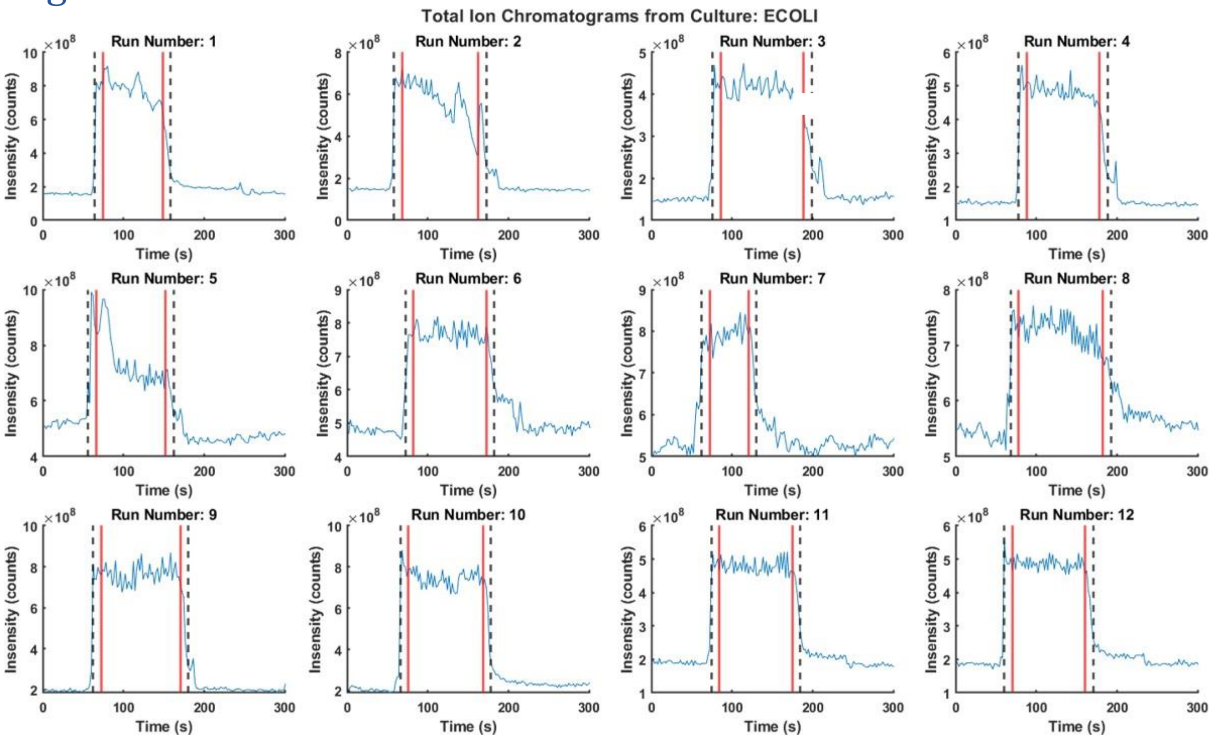

Figure S4: TIC response for each *Escherichia coli* headspace sample captured in a Tedlar bag.

Figure S5

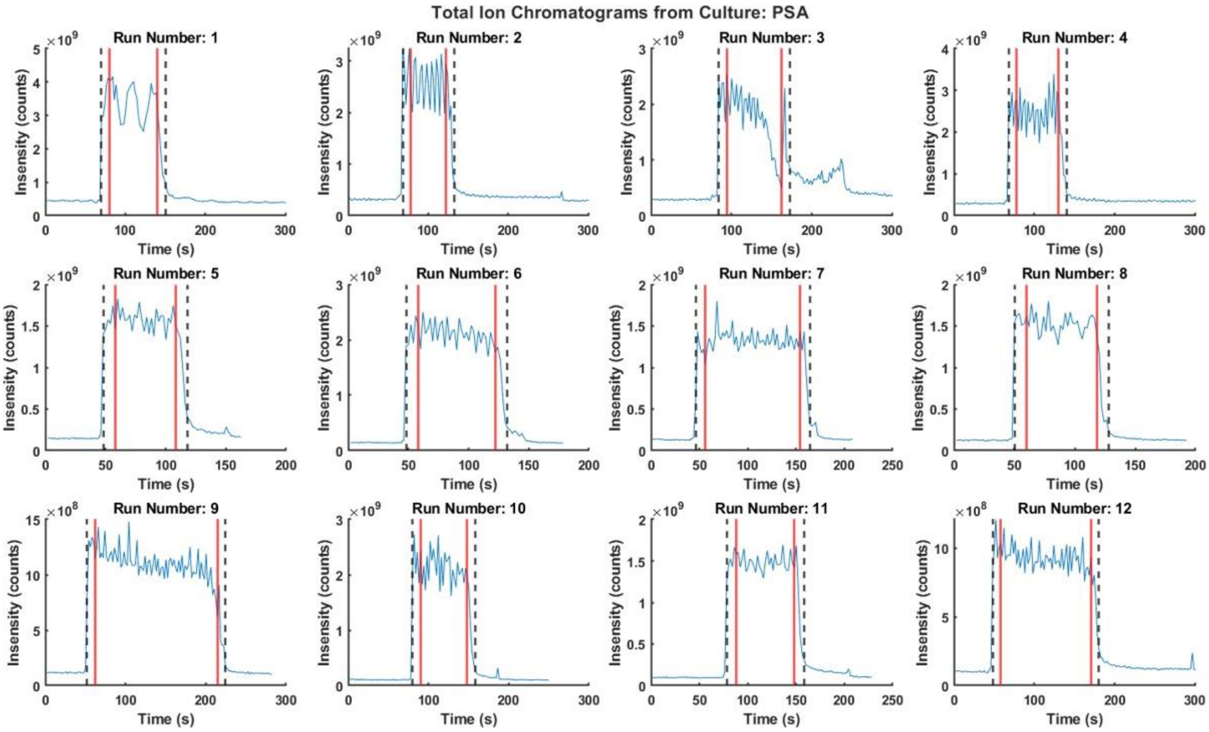

Figure S5: TIC response for each *Pseudomonas aeruginosa* headspace sample captured in a Tedlar bag.

Figure S6

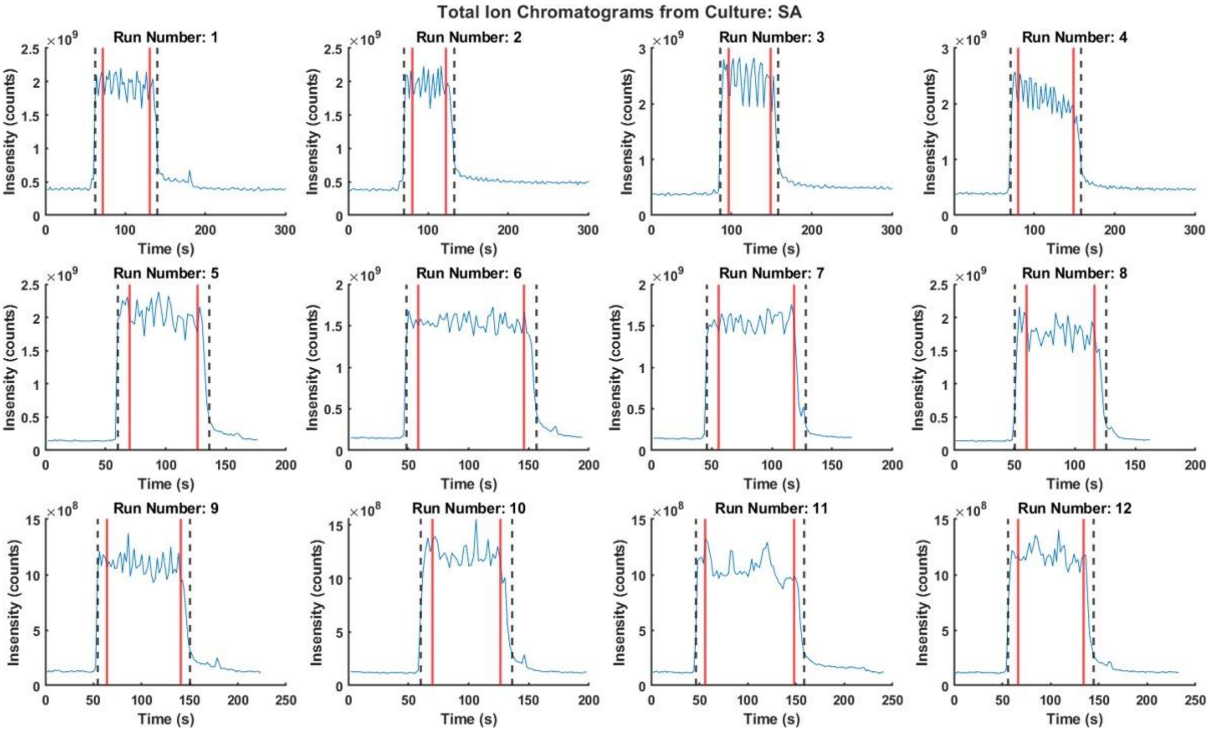

Figure S6: TIC response for each *Staphylococcus aureus* headspace sample captured in a Tedlar bag.

Figure S7

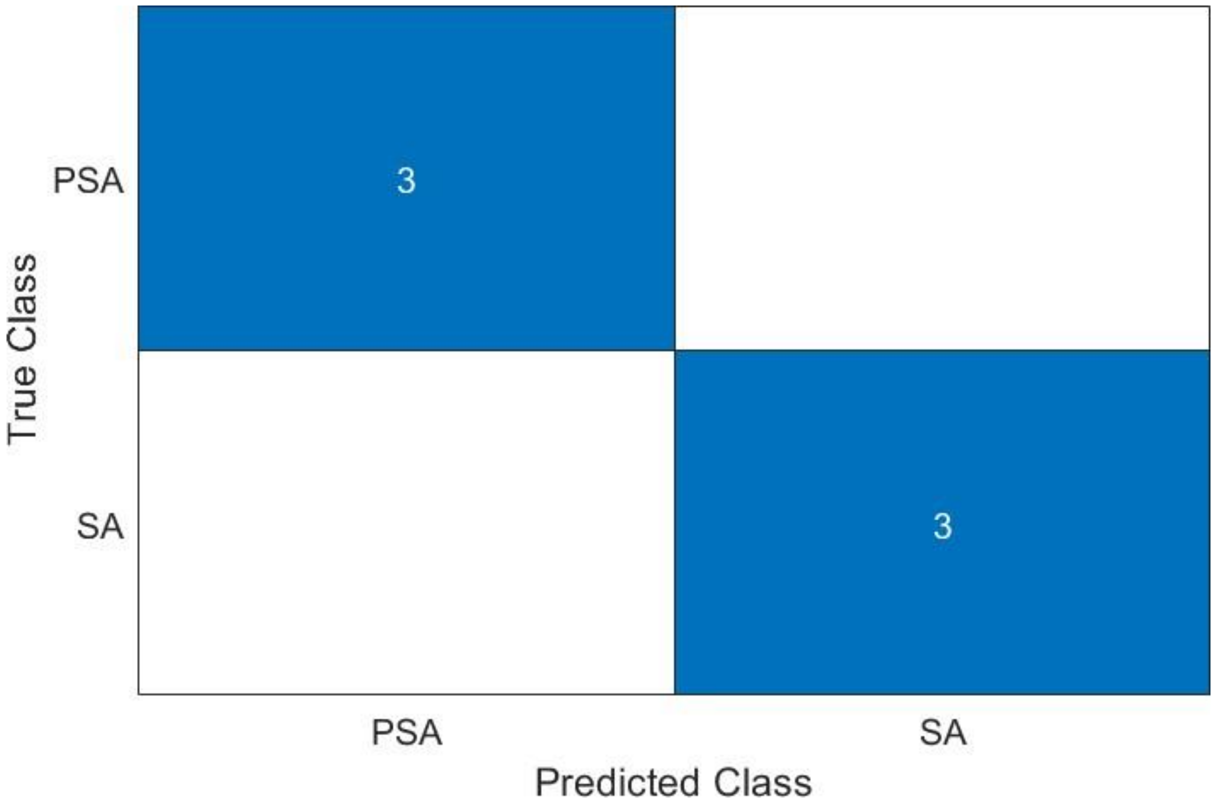

Figure S7: Predicted class versus true class for a blind test of three headspace samples from *Pseudomonas aeruginosa* and three headspace samples from *Staphylococcus aureus.*

Figure S8

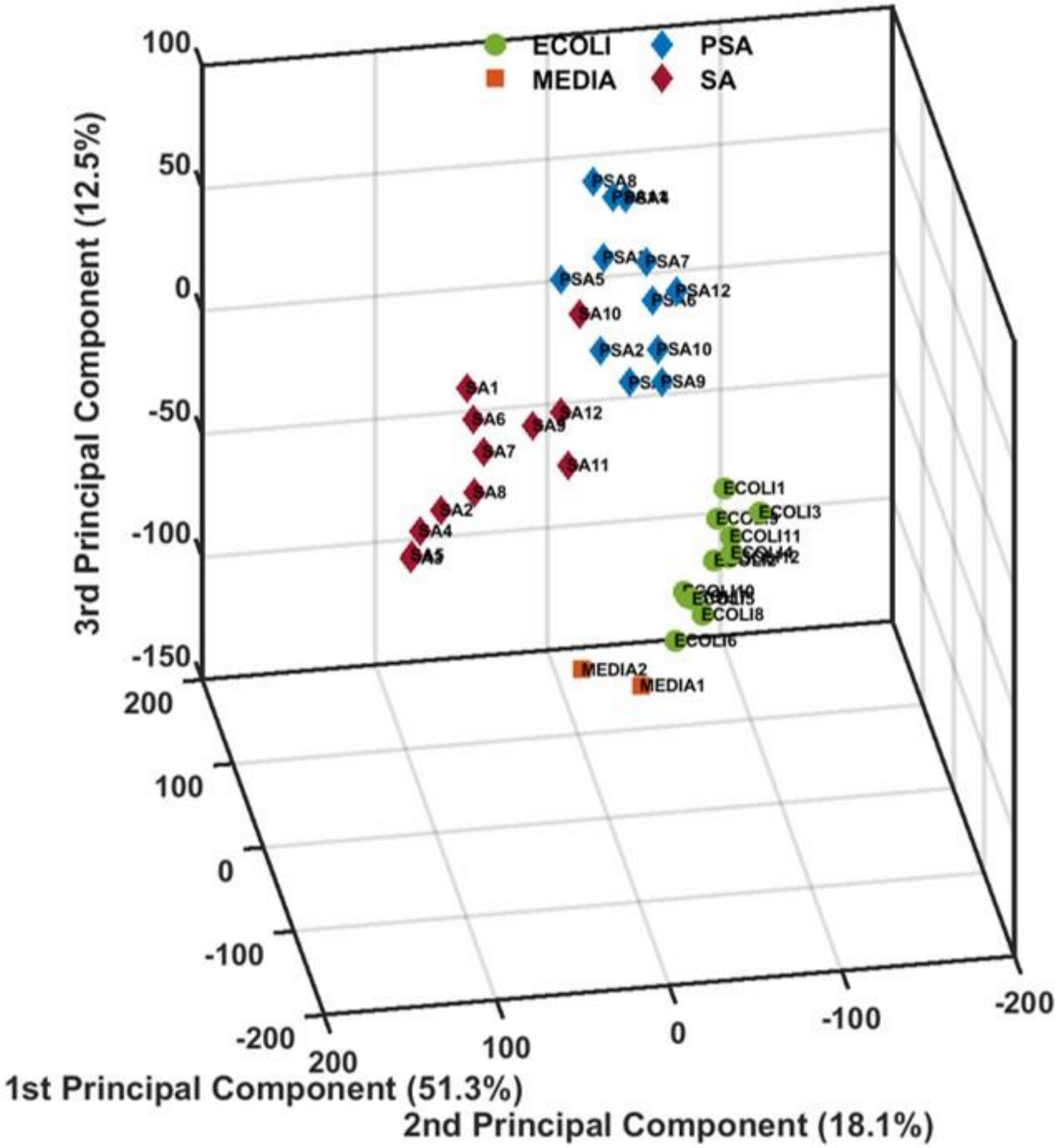

Figure S8: PCA plot showing the bacteria headspace samples, including 2 uninoculated broth samples.

Tedlar Bags: A Cautionary Note for Direct Analysis

A detailed literature survey indicated that Tedlar bags are the most common method for offline gas sampling in studies such as this. However, this work employs a direct analysis methodology without any pre-separation (e.g., GC). Thus, it is expedient to be mindful of any possible contamination and its implications.

Supplementary Figure S9 shows the spectral fingerprint for one PSA sample before and after the removal of known contaminants; the number of peaks visible after the removal of contaminants is startling, and further study is underway to examine alternative methods for gas collection that are less susceptible to contamination and thus provide a better representation of direct analysis. This issue is not expected in future direct breath analysis studies (once ethical approval has been obtained), as breath samples can be tested directly without the need for an intermediate collection step, significantly reducing the risk of contamination.

Figure S10 shows the result from injecting 2 μL of 20 ppm eucalyptol solution into a bag filled with nitrogen; we tested Tedlar bags from 2 suppliers, and both showed a very intense peak at m/z 88 suspected to be dimethylacetamide (DMAC) and a less intense dimer at m/z 175. This is a well-known contaminant from Tedlar bags.[3] The intensity of the DMAC peak from one supplier (Figure S10b) was sufficient to suppress the eucalyptol molecular ion peak to a background level; thus, for the collection of bacterial samples, we avoided using bags from this particular supplier. We note that though the contaminant peaks were still the most prominent in the spectra, they were not sufficiently intense to suppress the remaining analyte peaks entirely.

Figure S9

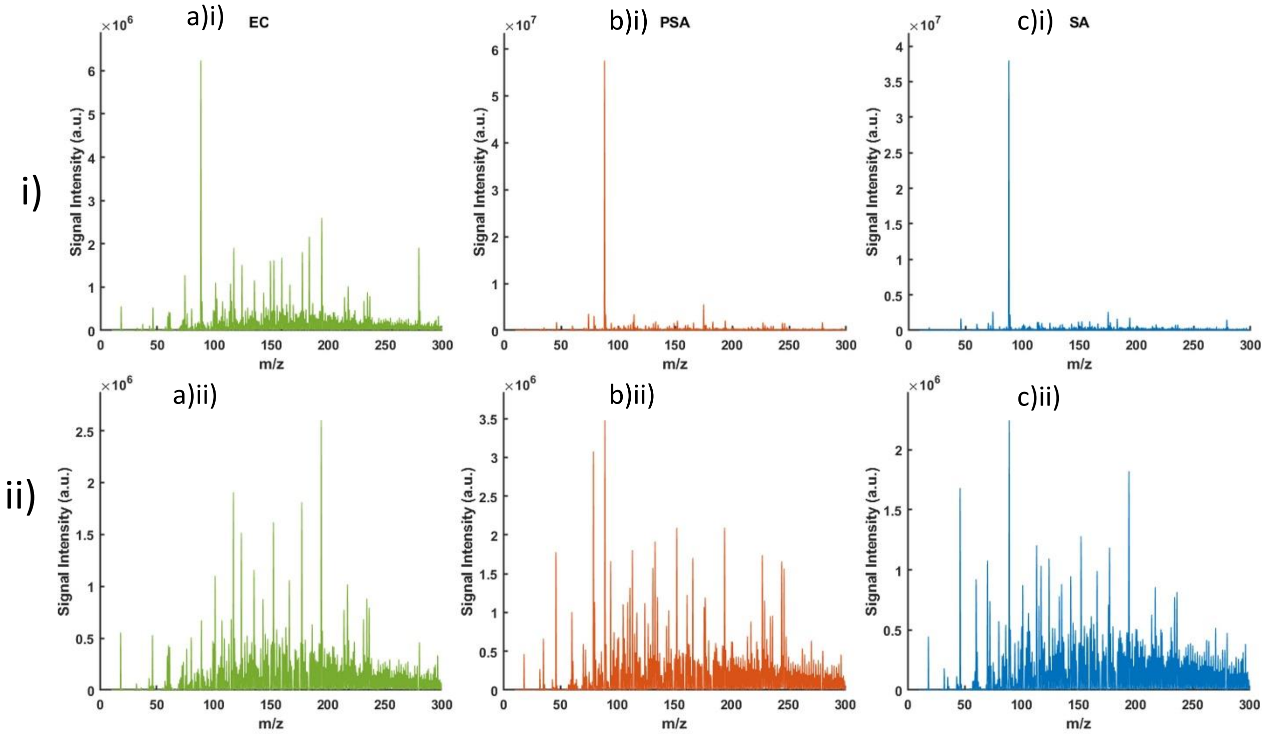

Figure S9: Mass spectra of (a) *Escherichia coli* (EC), (b) *Pseudomonas aeruginosa* (PSA), and (c) *Staphylococcus aureus* (SA) headspace samples, (i) before and (ii) after removal of known contaminants.

Figure S10

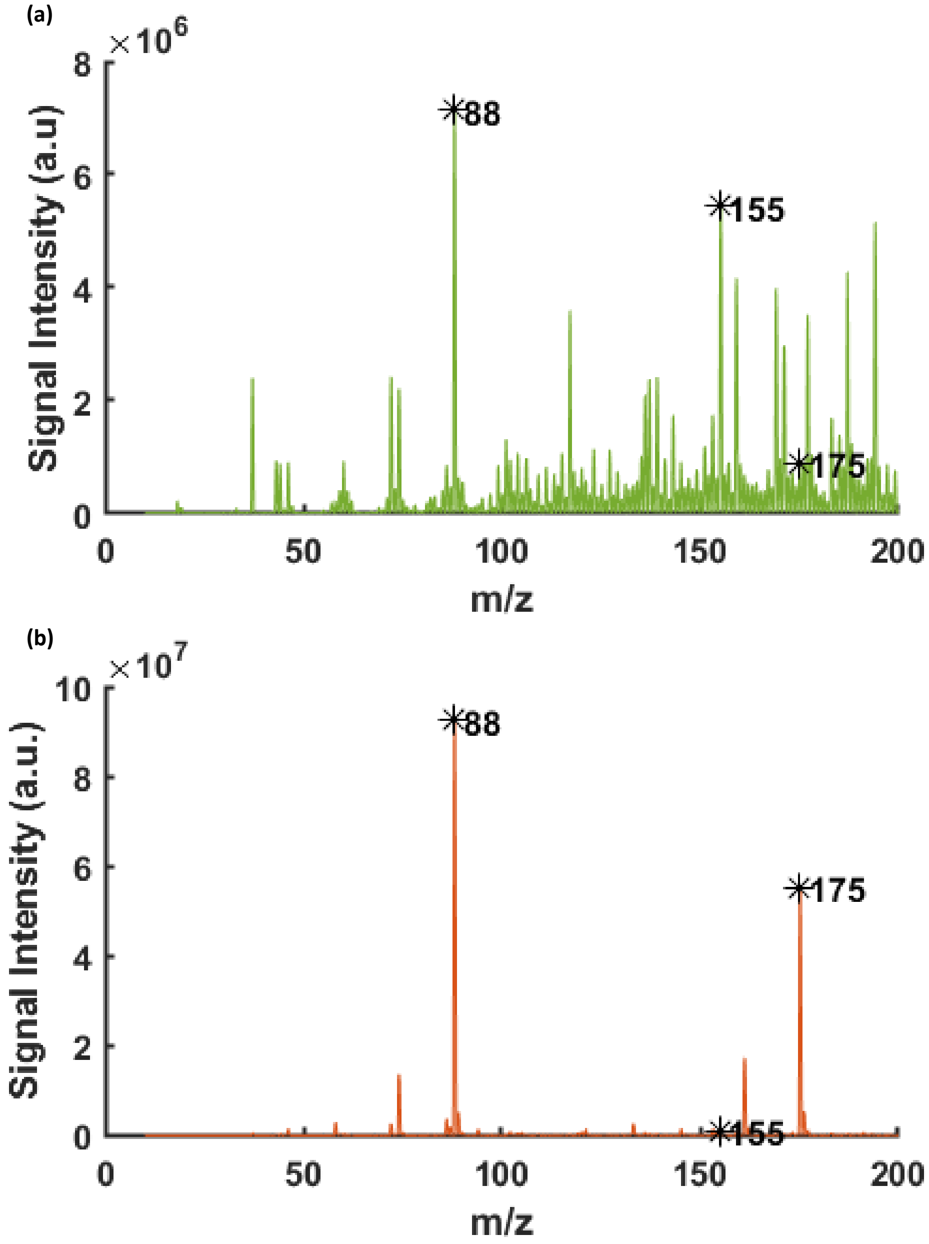

Figure S10: Mass spectra for 2 μL solution of 20 ppm eucalyptol (*m*/*z* 155) in water doped into 1 L Tedlar bags from two suppliers (a and b). *m*/*z* 88 and *m*/*z* 175 were suspected to be protonated dimethylacetamide, $[M + H]^+$ and its dimer, $[2M + H]^+$, respectively.

Figure S11

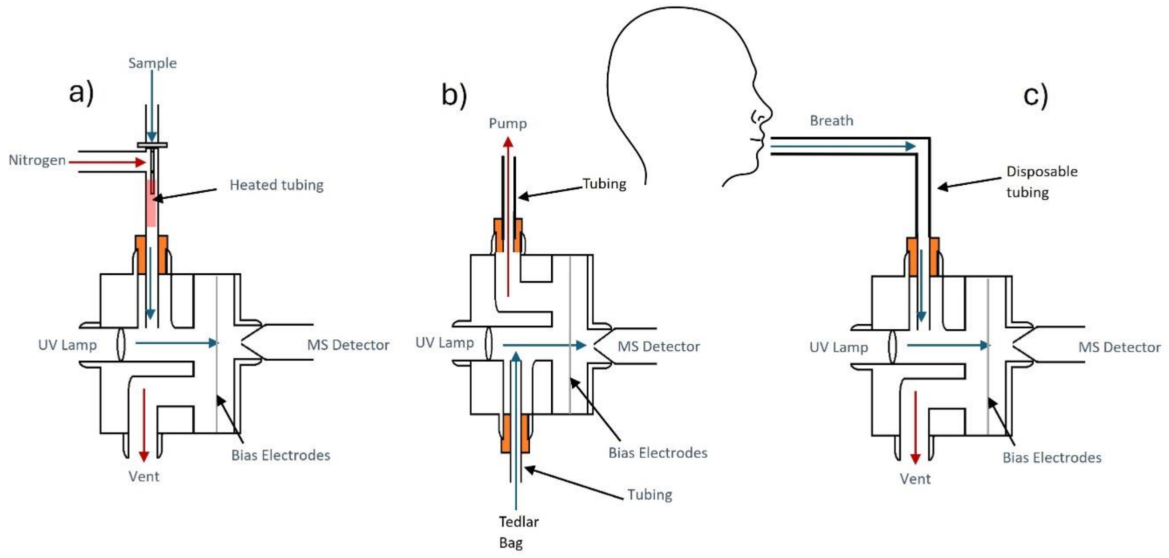

Figure S11: Schematic representation of the APPI sample interface apparatus for a) introduction of liquid phase standards, b) Tedlar bag evacuation using a small diaphragm pump and c) direct breath analysis.

Figure S12

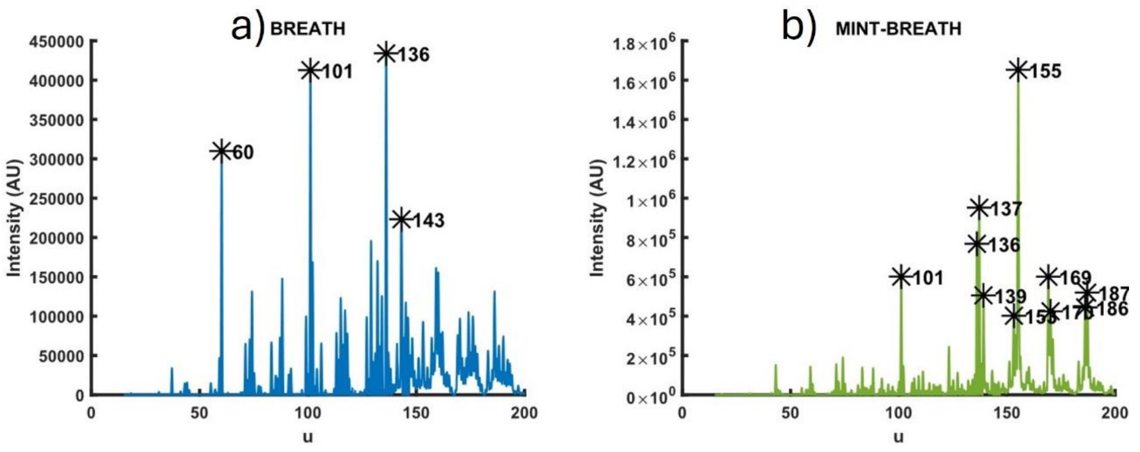

Figure S12: Mass spectra of volunteer breath a) before and b) after consuming mint chewing gum. Breath was sampled from a 1L Tedlar bag.

Table S1

Authors	Method	Bacteria	Sample	Volume	Sample time	Direct Sample	Separated scatterplot	Ref
K. F. Hintzen *et al* (2024)	TD-GC-ToF-MS	KP, PSA, SA	Headspace cultures	100 mL culture in 1 L flask	15 mins	No	No	[4]
W. Xu *et al* (2024)	PTR-MS,<br>FGC-PTR-MS	AB, ECL, EC, PA, SM, SA	Headspace cultures	25 mL culture in 125 mL Impinger, 100 mL HS flushed into PVF bags	10 mins	No	Yes	[5]
M. Woollam *et al* (2022)	SPME-GC-MS	N/a	Breath	3 L Tedlar bags	45 mins	No	N/a	[6]
K. Rosenthal *et al* (2021)	APCI-MS	EC, SA	Headspace cultures	5 mL agar volume in 20 mL vial	5 mins	Yes	No	[7]
M. Bannier *et al* (2019)	aeoNose	N/a	Breath	-	3 mins	Yes	No	[8]
C. Drees *et al* (2019)	NTD-GC-MS	EC, PSA, SA	Headspace cultures	1 L sample of 28 mL anaerobic or 38 mL aerobic culture.	100 mins	No	No	[9]
O. Lawal *et al* (2018)	TD-GC-MS	PA	Headspace cultures	1200 mL headspace sample	6 mins	No	N/a	[10]
O. Lawal *et al* (2018)	TD-GC-MS	ECL, PA	Headspace cultures	50 mL culture in 250 mL bottle	6 mins	No	Yes	[11]
M. Nasir *et al* (2018)	SPME-GCxGC-ToF-MS	PSA, SA	Headspace Bronchoalveolar	500 µL of BAL in 10 mL vial	60 mins	No	Yes	[12]

			lavage (BAL) fluid					
K. Dryahina *et al* (2016)	SIFT-MS	PSA, SA, SM, BCC	Headspace cultures	7 mL culture in 100 mL flask	3 mins	Yes	No	[13]
H. Li *et al* (2018)	SESI-MS	SA	Headspace cultures	15 mL culture in 100 mL bottle	1 min	Yes	N/a	[14]
V. Shestivska *et al* (2015)	SPME-GC-MS,<br>SIFT-MS	PSA	Headspace cultures	7 mL culture in 100 mL flask	30 mins (SPME-GC-MS)<br>4 mins (SIFT-MS)	Yes	N/a	[15]
A. Haworth-Duff *et al* (2024)	APPI-MS	EC, PSA, SA	Headspace cultures	1 L Tedlar bags	7 mins (2 mins sample collection, 5 mins analysis)	Yes	Yes	This study

Table S1: Comparison of some recent methods for headspace and breath sample analysis of some cystic fibrosis-related bacteria. Bacteria acronyms, EC = Escherichia coli, ECL = Enterobacter cloacae, KP = Klebsiella pneumoniae, PSA = Pseudomonas aeruginosa, SA = Staphylococcus aureus, SM = Stenotrophomonas maltophilia, BCC = Burkholderia cepacia complex. Other acronyms, APCI = Atmospheric pressure chemical ionisation, APPI = Atmospheric pressure photoionisation, FGC = Fast gas chromatography, GC = Gas chromatography, NTD = Needle trap device, MS = Mass spectrometry, PTR = Proton transfer reaction, SESI = Secondary electrospray ionisation, SIFT = Selected ion flow tube, SPME = Solid phase microextraction, TD = Thermal desorption, ToF = Time of flight.

The focus of this table was studies conducted in the last decade that analysed bacteria in either the headspace of cultures or from clinical breath samples. To indicate the current state of using VOCs produced by bacteria for detection, the survey focuses more on mass spectrometry techniques to aid comparison with our method.

Table S2

Analyte	Mw	Structure	Boiling point (°C)	Vapour Pressure (mmHg)	Ionisation Energy (eV)	Proton Affinity (kJ/mol)	Significance	Concentration range of interest	Ref.	APPI-MS LoD (ppbv)
Ethanol	46.07	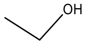	78.2	43.0	10.480	776.4	Background, lifestyle, renal failure	Healthy: 10-1000ppb	[16,17]	16.6
Acetone	58.08	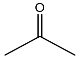	55.8	231.0	9.703	812.0	Background, diabetes	Healthy: 1-1000ppb<br>Diabetes: >1800ppb	[16,18-20]	6.8
2-Butanone	72.11	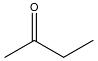	79.5	90.6	9.520	827.3	Background, lung cancer	0-26ppb	[21-23]	1.6
Ethyl Acetate	88.11	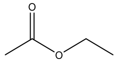	77.1	93.2	10.010	835.7	Lung disease	Healthy: 0ppb<br>Lung disease: 0-116ppb	[24]	0.7
Eucalyptol	154.25	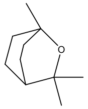	176.4	1.9	-	-	Lifestyle (consumption of mint)	-	[25,26]	0.9

Table S2: Analytes used in this study and their relevance to breath analysis, including therapeutic range approximations based on the literature.

Supplementary References

1 Kirsch, F. & Buettner, A. Characterisation of the metabolites of 1, 8-cineole transferred into human milk: Concentrations and ratio of enantiomers. *Metabolites* **3**, 47-71 (2013).

2 Horst, K. & Rychlik, M. Quantification of 1, 8-cineole and of its metabolites in humans using stable isotope dilution assays. *Molecular nutrition & food research* **54**, 1515-1529 (2010).

3 Trabue, S. L., Anhalt, J. C. & Zahn, J. A. Bias of Tedlar bags in the measurement of agricultural odorants. *J Environ Qual* **35**, 1668-1677, doi:10.2134/jeq2005.0370 (2006).

4 Hintzen, K. F. *et al.* Volatile organic compounds in headspace characterize isolated bacterial strains independent of growth medium or antibiotic sensitivity. *Plos one* **19**, e0297086 (2024).

5 Xu, W. *et al.* Qualitative and quantitative rapid detection of VOCs differentially released by VAP-associated bacteria using PTR-MS and FGC-PTR-MS. *Analyst* **149**, 1447-1454, doi:10.1039/d3an02011h (2024).

6 Woollam, M. *et al.* Preliminary method for profiling volatile organic compounds in breath that correlate with pulmonary function and other clinical traits of subjects diagnosed with cystic fibrosis: a pilot study. *Journal of Breath Research* **16**, 027103 (2022).

7 Rosenthal, K. *et al.* Volatile atmospheric pressure chemical ionisation mass spectrometry headspace analysis of E. coli and S. aureus. *Anal Methods* **13**, 5441-5449, doi:10.1039/d1ay01555a (2021).

8 Bannier, M., van de Kant, K. D. G., Jobsis, Q. & Dompeling, E. Feasibility and diagnostic accuracy of an electronic nose in children with asthma and cystic fibrosis. *J Breath Res* **13**, 036009, doi:10.1088/1752-7163/aae158 (2019).

9 Drees, C. *et al.* GC-IMS headspace analyses allow early recognition of bacterial growth and rapid pathogen differentiation in standard blood cultures. *Appl Microbiol Biotechnol* **103**, 9091-9101, doi:10.1007/s00253-019-10181-x (2019).

10 Lawal, O. *et al.* Volatile organic compound signature from co-culture of lung epithelial cell line with Pseudomonas aeruginosa. *Analyst* **143**, 3148-3155 (2018).

11 Lawal, O. *et al.* TD/GC-MS analysis of volatile markers emitted from mono- and co-cultures of Enterobacter cloacae and Pseudomonas aeruginosa in artificial sputum. *Metabolomics* **14**, 66, doi:10.1007/s11306-018-1357-5 (2018).

12 Nasir, M. *et al.* Volatile molecules from bronchoalveolar lavage fluid can 'rule-in' Pseudomonas aeruginosa and 'rule-out' Staphylococcus aureus infections in cystic fibrosis patients. *Sci Rep* **8**, 826, doi:10.1038/s41598-017-18491-8 (2018).

13 Dryahina, K., Sovova, K., Nemec, A. & Spanel, P. Differentiation of pulmonary bacterial pathogens in cystic fibrosis by volatile metabolites emitted by their in vitro cultures: Pseudomonas aeruginosa, Staphylococcus aureus, Stenotrophomonas maltophilia and the Burkholderia cepacia complex. *J Breath Res* **10**, 037102, doi:10.1088/1752-7155/10/3/037102 (2016).

14 Li, H. & Zhu, J. Differentiating antibiotic-resistant staphylococcus aureus using secondary electrospray ionization tandem mass spectrometry. *Analytical chemistry* **90**, 12108-12115 (2018).

15 Shestivska, V. *et al.* Quantification of methyl thiocyanate in the headspace of Pseudomonas aeruginosa cultures and in the breath of cystic fibrosis patients by selected ion flow tube mass spectrometry. *Rapid Commun Mass Spectrom* **25**, 2459-2467, doi:10.1002/rcm.5146 (2011).

16 Diskin, A. M., Spanel, P. & Smith, D. Time variation of ammonia, acetone, isoprene and ethanol in breath: a quantitative SIFT-MS study over 30 days. *Physiol Meas* **24**, 107-119, doi:10.1088/0967-3334/24/1/308 (2003).

17 Di Natale, C., Paolesse, R., Martinelli, E. & Capuano, R. Solid-state gas sensors for breath analysis: a review. *Anal Chim Acta* **824**, 1-17, doi:10.1016/j.aca.2014.03.014 (2014).

18 Righettoni, M. *et al.* Breath acetone monitoring by portable Si:WO3 gas sensors. *Anal Chim Acta* **738**, 69-75, doi:10.1016/j.aca.2012.06.002 (2012).
19 Anderson, J. C. Measuring breath acetone for monitoring fat loss: Review. *Obesity (Silver Spring)* **23**, 2327-2334, doi:10.1002/oby.21242 (2015).
20 Bajtarevic, A. *et al.* Noninvasive detection of lung cancer by analysis of exhaled breath. *BMC Cancer* **9**, 348, doi:10.1186/1471-2407-9-348 (2009).
21 Fu, X. A., Li, M., Knipp, R. J., Nantz, M. H. & Bousamra, M. Noninvasive detection of lung cancer using exhaled breath. *Cancer Med* **3**, 174-181, doi:10.1002/cam4.162 (2014).
22 Fenske, J. D. & Paulson, S. E. Human breath emissions of VOCs. *J Air Waste Manag Assoc* **49**, 594-598, doi:10.1080/10473289.1999.10463831 (1999).
23 Van den Velde, S., Nevens, F., Van Hee, P., van Steenberghe, D. & Quirynen, M. GC-MS analysis of breath odor compounds in liver patients. *J Chromatogr B Analyt Technol Biomed Life Sci* **875**, 344-348, doi:10.1016/j.jchromb.2008.08.031 (2008).
24 Khatoon, Z. *et al.* Ethyl Acetate Chemical Sensor as Lung Cancer Biomarker Detection Based on Doped Nano-SnO_2 Synthesized by Sol-Gel Process. *IEEE Sensors Journal* **20**, 12504-12511, doi:10.1109/JSEN.2020.3001285 (2020).
25 Ruszkiewicz, D. M. *et al.* Peppermint protocol: first results for gas chromatography-ion mobility spectrometry. *J Breath Res* **16**, doi:10.1088/1752-7163/ac6ca0 (2022).
26 Lu, Y. *et al.* Glass bottle sampling solid phase microextraction gas chromatography mass spectrometry for breath analysis of drug metabolites. *J Chromatogr A* **1496**, 20-24, doi:10.1016/j.chroma.2017.03.061 (2017).
